# Spatiotemporal distribution and fluctuation of radiocesium in Tokyo Bay in the five years following the Fukushima Daiichi Nuclear Power Plant (FDNPP) accident

**DOI:** 10.1371/journal.pone.0193414

**Published:** 2018-03-01

**Authors:** Hideo Yamazaki, Masanobu Ishida, Ryoichi Hinokio, Yosuke Alexandre Yamashiki, Ryokei Azuma

**Affiliations:** 1 Graduate School of Science and Engineering, Kindai University, Higashiosaka, Osaka, Japan; 2 Faculty of Science and Technology, Ryukoku University, Otsu, Shiga, Japan; 3 Graduate School of Advanced Integrated Studies in Human Survivability, Kyoto University, Kyoto, Kyoto, Japan; 4 Department of Civil Engineering and Urban Design, Osaka Institute of Technology, Osaka, Osaka, Japan; University of South Carolina, UNITED STATES

## Abstract

A monitoring survey was conducted from August 2011 to July 2016 of the spatiotemporal distribution in the 400 km^2^ area of the northern part of Tokyo Bay and in rivers flowing into it of radiocesium released from the Fukushima Daiichi Nuclear Power Plant (FDNPP) accident. The average inventory in the river mouth (10 km^2^) was 131 kBq⋅m^-2^ and 0.73 kBq⋅m^-2^ in the central bay (330 km^2^) as the decay corrected value on March 16, 2011. Most of the radiocesium that flowed into Tokyo Bay originated in the northeastern section of the Tokyo metropolitan area, where the highest precipitation zone of ^137^Cs in soil was almost the same level as that in Fukushima City, then flowed into and was deposited in the Old-Edogawa River estuary, deep in Tokyo Bay. The highest precipitation of radiocesium measured in the high contaminated zone was 460 kBq⋅m^-2^. The inventory in sediment off the estuary of Old-Edogawa was 20.1 kBq⋅m^-2^ in August 2011 immediately after the accident, but it increased to 104 kBq⋅m^-2^ in July 2016. However, the radiocesium diffused minimally in sediments in the central area of Tokyo Bay in the five years following the FDNPP accident. The flux of radiocesium off the estuary decreased slightly immediately after the accident and conformed almost exactly to the values predicted based on its radioactive decay. Contrarily, the inventory of radiocesium in the sediment has increased. It was estimated that of the 8.33 TBq precipitated from the atmosphere in the catchment regions of the rivers Edogawa and Old-Edogawa, 1.31 TBq migrated through rivers and was deposited in the sediments of the Old-Edogawa estuary by July 2016. Currently, 0.25 TBq⋅yr^-1^ of radiocesium continues to flow into the deep parts of Tokyo Bay.

## Introduction

Tokyo Bay is a closed bay that extends 70 km from north to south and 20 km from east to west, covers a total area of 1,380 km^2^, is an average of 15 m deep, and is connected to the Pacific Ocean by a 7 km wide strait at its south end. The average retention time of seawater varies seasonally but is reported to be approximately 31 days [1]. Central Tokyo is located on the west side of the bay, which is surrounded by a zone of large cities that forms the heart of Japan, and has a total population of 38 million. The catchment basins of rivers flowing into Tokyo Bay from the greater Tokyo region occupy a land area of 9,100 km^2^, and the total quantity of inflowing river water fluctuates greatly, but averages approximately 1.4 × 10^7^ m^3^⋅day^-1^. The major rivers are the Edogawa, Old-Edogawa, Arakawa, Tamagawa, Sumidagawa, and Tsurumigawa. Even though Tokyo Bay is closed, its seawater flow is complex. In addition to tidal currents, permanent currents flow throughout the bay, and the surface water movement is dominated by circular drifts: clockwise in the winter and counterclockwise in the summer. The bottom water moves in the opposite direction to the flow of the surface water. The pelagic water from the Pacific Ocean flows north on the bottom inside the bay until it reaches the Bay’s deepest section [1].

Aircraft monitoring of the ^134+137^Cs precipitation was conducted by the Ministry of Education, Culture, Sports, Science and Technology of Japan (MEXT) [2], and the results were published by the Geospatial Information Authority of Japan (GSI) [3], showing that the catchment basin of Edogawa River was contaminated from 30 to 100 kBq⋅m^-2^ by radiocesium discharged from the Fukushima Daiichi Nuclear Power Plant (FDNPP) accident, but the radioactive contamination levels in the catchment basins of the Bay’s other rivers were lower than those in the catchment basin of Edogawa. Radioactive materials precipitated on the ground surface in the greater Tokyo region are, as in the case of artificially discharged environmental contaminants, presumably carried by these rivers until they finally flow into Tokyo Bay.

Many reports outlining the FDNPP accident have already been released to the public [4–7]. However, many of these are analyses of the accident process, whereas few address the environmental radioactive contamination that was caused [2, 8–10]. In particular, the movement in the greater Tokyo region of radioactive contamination produced by the FDNPP accident has been insufficiently analyzed. Nevertheless, after the accident, high concentration radioactive plumes arrived in the greater Tokyo region, radionuclides washout with precipitation (rainfall) on March 16 and 22 in 2011 [11]. Clarifying the movement of environmental radioactive contaminants in the heavily populated greater Tokyo region is an important issue related to the problem of low dose exposure to large populations. Evaluation of the migration process of radiocesium from the Tokyo metropolitan area also is important from the viewpoint of reduction and decontamination of radioactive contamination in these areas. In our previous paper, the behavior of radioactive contaminants of the soil in the Tokyo metropolitan area was discussed [12]. It is estimated that 10 to 22% of the radiocesium precipitated in the surface soil and migrated to Tokyo Bay via rivers in the five years after the FDNPP accident.

This study was a continuous time-series analysis of the distribution and fluctuation of radiocesium in sediments and waters in Tokyo Bay and in the rivers flowing into Tokyo Bay starting in August 2011, immediately after the FDNPP accident. Based on the results, the process of the movement from the land and deposition in Tokyo Bay of radiocesium that was precipitated in the greater Tokyo region via the FDNPP accident were evaluated. However, before the FDNPP accident the Chernobyl accident and the Three Mile Island (TMI) accident affected many affecting people. TMI was located about 150 km west of Washington DC but because it avoided the destruction of the pressure vessel, the emission of radioactive nuclides to the environment is only rare gas nuclides, and the release amount of ^131^I is estimated to be about 0.5 TBq [13,14]. In the case of the Chernobyl accident, Kiev City was located 130 km south, and 4 million residents lived in that metropolitan area. It was reported at the Chernobyl Forum by the IAEA in 2006 [15] that radioactive plume flew to Kyiv City by the north wind on May 1, 1986 immediately after the accident. However, regulations on information disclosure were made by the Soviet government at the time, and the actual state and dynamics of radioactive contamination in Kiev City are still hardly understood even now. Of course, do not forget the radioactive contamination by the atomic bombs of Hiroshima and Nagasaki. The results of our investigation on the environmental dynamics of 60 years after radiation exposure in Nagasaki has already been reported [16–18]. From such a viewpoint, we think that the FDNPP accident was the first time that an urban region as densely populated as Tokyo was contaminated by radioactive material over a wide area.

Studies on the behavior of radiocesium in an urban environment have been performed through simulations [19], but fluctuation in this radionuclide’s spatiotemporal distribution has not been monitored nor analyzed over wide areas for long periods. Furthermore, the behavior of cesium as an alkaline element is often complicated and unknown in the estuary where seawater and river water mix [20,21]. In this study, the important roles that Tokyo Bay and rivers flowing into it play in the movement of radiocesium contaminants and the transport and accumulation mechanisms of such in the greater Tokyo region have been clarified.

The Nuclear Regulation Authority of the Japanese Government (NRA) has monitored the radioactive contamination derived from the FDNPP accident in the surface sediment of Tokyo Bay since June 2013 [22]. The Japan Coast Guard (JCG) has also been measuring the radioactive contamination of surface sediments in Tokyo Bay since 1981 [23]. On the other hand, survey results of radiocesium contamination in the Tokyo Bay area immediately after the accident have been published [24]. However, since their monitoring is limited spatiotemporally, it is insufficient to evaluate the dynamics of radioactive contamination throughout the environments of Tokyo Bay.

## Sampling and analytical methods

### Material and methods

**Fig 1:**
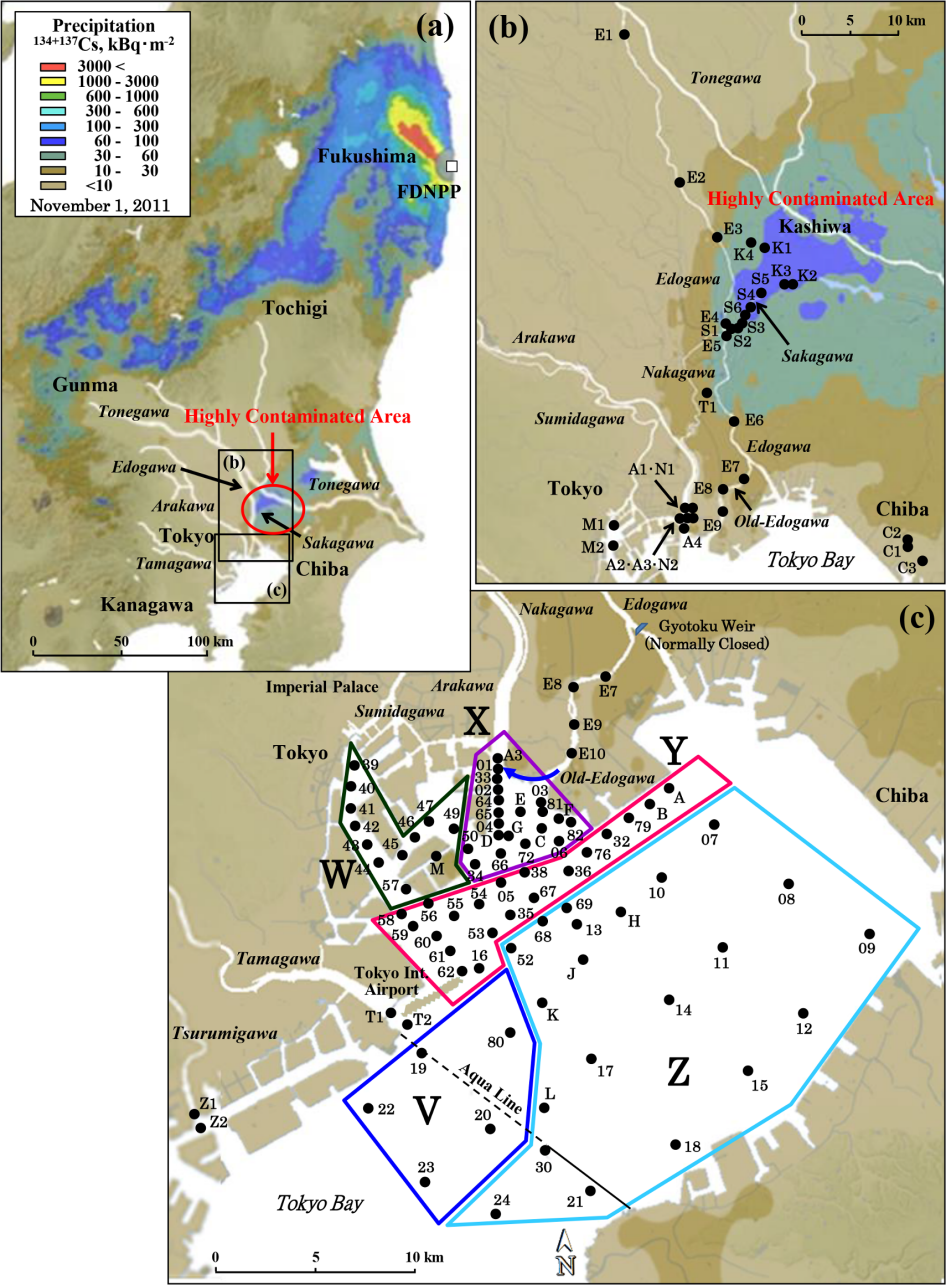
Study areas and sampling points. Geographical distribution of the radiocesium precipitation is indicated by the values for eight months after the accident, adapted from “Extension Site of Distribution Map of Radiation Dose, etc.” [3]. (a) Study area. (b) Sampling points in the Edogawa river system. (c) Sampling points in the Tokyo Bay area. V: Tamagawa estuary, W: Sumidagawa estuary, X: Old-Edogawa estuary, Y: Off the Old-Edogawa estuary, Z: Center of Tokyo Bay, Aqua Line: Cross road of Tokyo Bay. River water in Old-Edogawa flows in the direction of the blue arrow in Fig 1C.

Sediment and water were sampled in Tokyo Bay and in the rivers in its catchment basin. The locations are shown in Fig 1. Sampling was performed at the same points one to seven times during the study period, which ran from August 20, 2011, until July 12, 2016. Sediment samples were collected at 77 points in Tokyo Bay, 10 points in Edogawa River, and 6 points in Sakagawa River. Of the sediment samples collected, 68 were core sediments and 142 surface sediments. Sediment cores were sampled at Point S1 (Fig 1), where Sakagawa flows into Edogawa, to evaluate the role of Sakagawa in the process of transporting radiocesium. To compare with sediment, soil samples were also collected from the 14 points shown in Fig 1B.

Sediment core sampling was done using an acrylic pipe with a diameter of 10 cm and length of 100 cm. The core samples were collected by a diver pushing the pipe into the seabed by hand. Core samples of 20 to 80 cm length were obtained. Surface sediment specimens were sampled from a boat using an Ekman-Birge bottom sampler. Then, on the boat, after the sediments were collected, the material was inserted into an acrylic pipe with a diameter of 5 cm and length of 10 cm to obtain samples a top 5 cm sediment. Most of soil and sediment samples consisted of silt and sand with a particle size of 2 mm or less. However, more pebbles, plant pieces, shell fragments, etc. were removed with tweezers. Grain size sorting by sieve was not done. The sediments were pushed out of the pipes, cut into 1 or 2 cm thick slices in the depth direction, and then thermally dried to a constant weight in a 60°C oven to remove the water from the sediments. The dried samples were pulverized in an agate mortar, and then the radioactivity of the samples was measured. Water samples were obtained from the surface of the water by lowering buckets from boats. Divers obtained bottom water from about 1 m above the seabed. Without filtering suspended materials out of the water, the radiocesium in 20 L of sample water was concentrated using an ammonium phosphomolybdate (AMP) method [25]. After standing overnight, the AMP precipitate was filtrated and collected on a membrane filter (pore size 0.8 μm), then the radioactivity of the dried AMP precipitate was measured. In this way, it was confirmed in a preliminary experiment that the ionic and suspended radiocesium in the sample water can be recovered quantitatively.

### Measurement of radioactivity

Radionuclides in the samples were quantified by connecting a 4096-multichannel pulse height analyzer (Lab Equipment, MCA600) to a low energy HPGe detector (ORTEC, LO-AX/30P) shielded in lead 10 cm thick, sealing the specimens inside a plastic container with a diameter of 5.5 cm and depth of 2.0 cm, then measuring them via γ-ray spectrometry. The Ge detector calculated the geometric efficiency relative to the sample weight using the American NIST (National Institute of Standards and Technology) Environmental Radioactivity Standards, SRM 4350B (River Sediment) and SRM 4354 (Freshwater Lake Sediment), and the efficiency was corrected to within a range of 2 to 30 g of the sample weight [26]. The measurement time was set so that the counting error would be less than 5% according to the radioactive intensity of the samples. ^134^Cs (605 keV) and ^137^Cs (662 keV) were quantified in this study. A ^134^Cs solution with known concentration was used to correct the sum peak effect for ^134^Cs counting. The detection limits of ^134^Cs and ^137^Cs under appropriate conditions were 0.6 Bq⋅kg^-1^ in sediment samples and 0.3 mBq⋅L^-1^ in water samples. Radiocesium activity was indicated by the values per sampling day, but was corrected for radioactive decay to the value on March 16, 2011, as necessary. In that case, it is denoted as “corrected activity.”

### Measurements of heavy metals and particle size distributions in the sediments

The heavy metals in the sediments were measured via an XRF method (Rigaku, ZSX-Primus Ⅱ) using the NIST SRM 1646 (Estuarine Sediment) as the standard sample. Sample measured were made from cellulose powder pressed into 4 cm diameter aluminum ring 0.4 ton⋅cm^-2^, and then 1.2 g of powdered sample was placed on the disk and repressed at 1.6 ton⋅cm^-2^. The correction of matrix effect was achieved by X-ray intensity ratio of peak to back ground for each element [27]. Mercury in the sediments was measured via a heating-vaporization atomic absorption spectrometry (Hiranuma, HG-300). The particle size distribution of the wet sediment samples was measured using a laser diffractometer (Shimadzu, SALD-3000) with a measurement range of particle size 0.05 to 3000 μm. Dispersion of sedimentary particles was carried out via ultrasonic irradiation (Shimadzu, SUS-200, 42 kHz) using sodium hexametaphosphate as a dispersant. In this paper, the particle size obtained is presented as the volume-based average particle diameter.

## Results

### Spatiotemporal distribution of radiocesium in Tokyo Bay sediment

**Fig 2:**
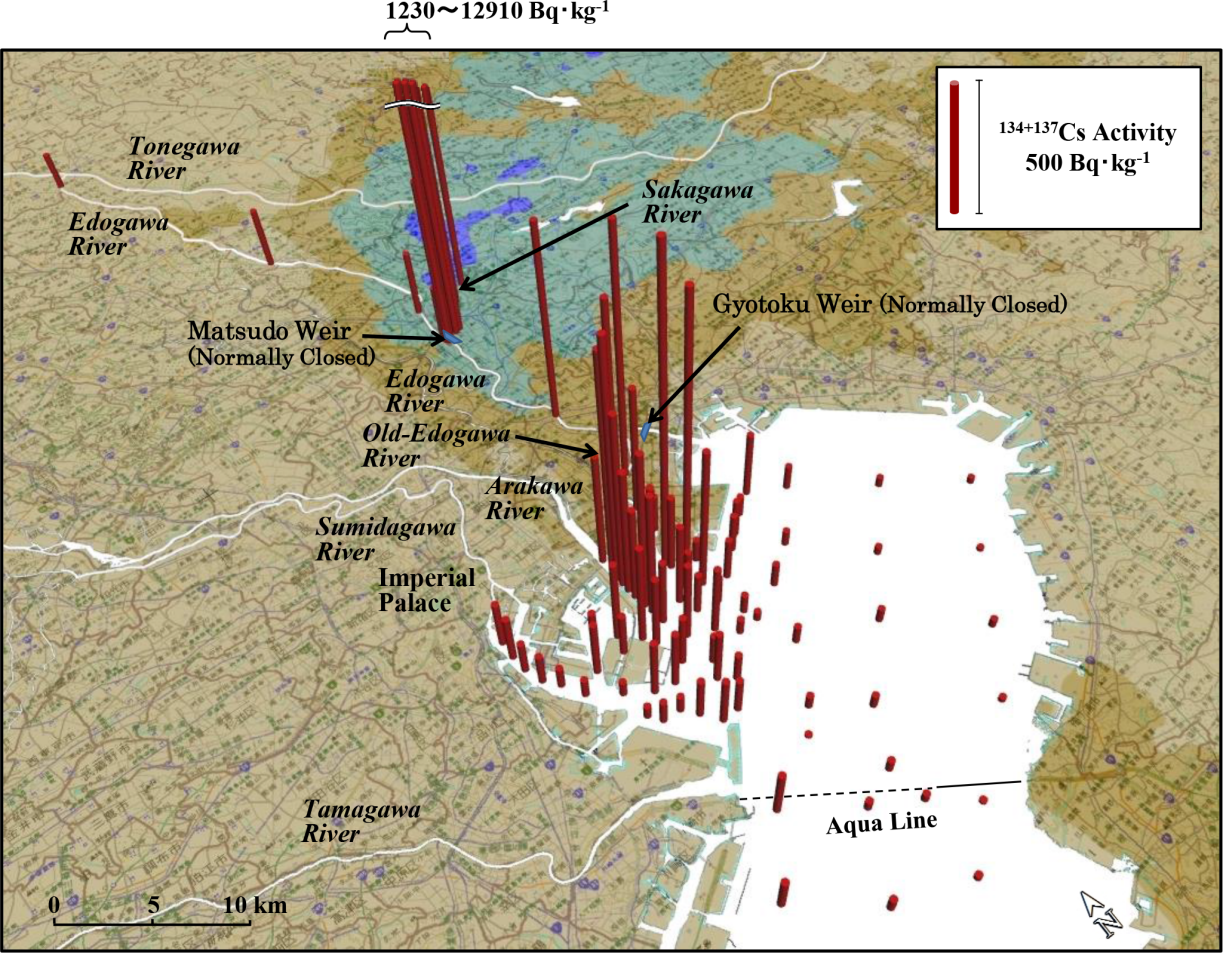
Activities of ^134+137^Cs in the surface sediments throughout the Tokyo Bay water system. Sediment samples were collected from August 20, 2011, to July 12, 2016. The activity of ^134+137^Cs was radioactive decay corrected based on the value of March 16, 2011. The value of activity is shown as an average of the values from the surface to 5 cm depth. When there are multiple data at the same point, the activity is expressed as a weighted average value for the counting error.

All measured values obtained in this study are shown in S1–S4 Tables of the supporting information file. Geographic coordinates of sampling points are also shown in S5 Table. Sampling was done on different days; hence, the radiocesium activities are shown after the radioactive decay correction based on the value of March 16, 2011. A plurality of measured values collected at different times were subjected to statistical processing. Fig 2 (S6 Table) shows the spatial distribution of the ^134+137^Cs activity (total value of ^134^Cs and ^137^Cs) in the surface layer of the sediment, from 0 to 5 cm depth. When multiple measurements were done at the same point, the ^134+137^Cs activity was evaluated based on the value in a weighted average with the counting error. The deviation of the weighted average approximated according to the law of uncertainty propagation.

The highest ^134+137^Cs activity among all measured values in Tokyo Bay was 1340 ± 13 Bq⋅kg^-1^, found in the surface sediments sampled at Point 01 in the Old-Edogawa estuary on November 1, 2012. As shown in Fig 2, the ^134+137^Cs activity in surface sediment in Tokyo Bay was ranked from high to low contamination level as the Old-Edogawa estuary (X), off the Old-Edogawa estuary (Y), center of Tokyo Bay (Z), Tamagawa estuary (V), and Sumidagawa estuary (W). Throughout the survey period, the ^134+137^Cs activity of the surface sediments was highest in the Area X, and fell remarkably towards the Area Z. The ^134+137^Cs activity indicated by the weighted average value was 424 ± 1 Bq⋅kg^-1^ (78–1340 Bq⋅kg^-1^, n = 55) for X, 131 ± 1 Bq⋅kg^-1^ (40–371 Bq⋅kg^-1^, n = 40) for Y, and 17 ± 0.3 Bq⋅kg^-1^ (1–162 Bq⋅kg^-1^, n = 50) for Z. In the Tamagawa estuary (V), which flows through farmland in western metropolitan Tokyo, the weighted average activity was 57 ± 1 Bq⋅kg^-1^ (5–234 Bq⋅kg^-1^, n = 7). In the Sumidagawa mouth (W), which flows through central Tokyo, it was 103 ± 2 Bq⋅kg^-1^ (32–374 Bq⋅kg^-1^, n = 16). Both are lower activities than that of the Old-Edogawa estuary (X).

We inferred the inventory and flux of radiocesium accumulation in Tokyo Bay sediment from the catchment basin owing to the FDNPP accident for the five years studied. Table 1 shows the inventory, flux, and ^134^Cs/^137^Cs activity ratio of radiocesium in the sediments collected from the Edogawa water system and Tokyo Bay. The ^134^Cs/^137^Cs activity ratio in 117 sediment samples (Table 1, S6 Table), with counting error of the radioactivity measurements within 5%, was 1.006 ± 0.003 (weighted average value), which conforms to the ^134^Cs/^137^Cs ratio of radiocesium discharged by the FDNPP accident [28–30].

**Table 1:** Inventory and flux of radiocesium in the sediment of the Tokyo Bay water system.

Area		Point	Sampling Date	Elapsed Time, day	Contami-nated Layer, cm	Inventory, kBq⋅m^-2^									Flux, kBq⋅m^-2^day^-1^			Activity Ratio ^134^Cs/^137^Cs
						^134^Cs			^137^Cs			^134+137^Cs			Total			
Edogawa River		E1	2011/12/3	262	<5	3.5	±	0.2	4.2	±	0.2	7.6	±	0.2	0.033	±	0.001	1.039
			2015/11/11	1701	25	22.3	±	0.4	95.7	±	0.9	118	±	1	0.126	±	0.001	1.005
		E2	2011/12/3	262	<5	6.2	±	0.2	7.6	±	0.2	13.8	±	0.3	0.060	±	0.001	1.031
			2015/11/12	1702	15	2.5	±	0.2	10.5	±	0.3	13.0	±	0.3	0.014	±	0.000	1.012
		E3	2011/12/3	262	<5	15.3	±	0.3	18.5	±	0.3	33.9	±	0.5	0.146	±	0.002	1.036
			2015/11/12	1702	5	0.3	±	0.1	1.4	±	0.1	1.8	±	0.1	0.002	±	0.000	1.027
		E4	2011/12/3	262	<5	12.1	±	0.3	15.2	±	0.3	27.4	±	0.4	0.118	±	0.002	0.998
			2015/11/12	1702	24	1.7	±	0.2	7.1	±	0.3	8.8	±	0.4	0.009	±	0.001	1.026
		E5	2011/12/3	262	<5	10.7	±	0.3	13.2	±	0.3	23.9	±	0.5	0.103	±	0.002	1.023
			2014/3/24	1104	<5	15.9	±	0.3	39.5	±	0.5	55.4	±	0.6	0.078	±	0.001	1.036
			2015/11/12	1702	20	1.6	±	0.2	7.3	±	0.3	8.9	±	0.4	0.009	±	0.001	0.959
		E6	2011/12/3	262	<5	27.5	±	0.5	34.4	±	0.6	61.9	±	0.8	0.267	±	0.003	1.000
			2015/11/12	1702	31.5	29.4	±	0.6	127	±	1	156	±	1	0.166	±	0.002	1.003
		E7	2011/12/3	262	<5	23.8	±	0.4	31.5	±	0.5	55.4	±	0.7	0.238	±	0.003	0.947
			2014/3/24	1104	<5	2.5	±	0.1	6.3	±	0.2	8.8	±	0.3	0.012	±	0.000	1.027
		E8	2012/4/3	384	<5	8.3	±	0.3	11.1	±	0.3	19.3	±	0.5	0.060	±	0.001	1.038
		E9	2012/4/3	384	<5	8.3	±	0.3	11.6	±	0.3	19.9	±	0.4	0.062	±	0.001	1.000
		E10	2012/4/3	384	<5	3.1	±	0.2	4.6	±	0.2	7.7	±	0.3	0.024	±	0.001	0.949
Sakagawa		S5	2014/4/29	1140	13.2	175	±	2	462	±	3	637	±	3	0.874	±	0.005	1.009
		S4	2014/4/29	1140	14	56.4	±	0.7	151	±	1	207	±	1	0.284	±	0.002	0.996
		S6	2014/4/29	1140	41	213	±	3	553	±	3	766	±	5	1.055	±	0.008	1.023
		S3	2014/4/29	1140	5	31.9	±	0.4	85.3	±	0.7	117	±	1	0.160	±	0.001	0.994
		S2	2014/4/29	1140	14	87.8	±	0.9	234	±	1	321	±	2	0.440	±	0.003	1.001
		S1	2014/4/29	1140	50	400	±	2	1050	±	3	1450	±	4	1.997	±	0.006	1.011
			2015/11/13	1703	12	13.5	±	0.3	57.6	±	0.6	71.2	±	0.7	0.076	±	0.001	1.012
			2016/6/25	1928	32	50.5	±	0.7	257	±	2	307	±	2	0.305	±	0.002	1.029
Tokyo Bay	X	02	2012/11/1	596	12	8.5	±	0.1	13.9	±	0.2	22.4	±	0.2	0.049	±	0.000	1.024
			2016/7/12	1945	52	14.0	±	0.3	74.7	±	0.6	88.7	±	0.7	0.087	±	0.001	0.995
		64	2013/10/4	933	26	11.2	±	0.2	24.7	±	0.3	35.9	±	0.3	0.056	±	0.001	1.009
		D	2011/8/20	157	26	9.3	±	0.2	10.8	±	0.2	20.1	±	0.2	0.138	±	0.002	0.984
			2012/4/2	383	26	12.1	±	0.1	17.0	±	0.1	29.1	±	0.2	0.090	±	0.001	0.986
			2012/11/1	596	44	21.0	±	0.2	34.7	±	0.3	55.8	±	0.4	0.122	±	0.001	1.012
			2013/10/4	933	44	17.7	±	0.2	39.2	±	0.3	56.9	±	0.4	0.089	±	0.001	1.007
			2014/3/25	1105	42	17.2	±	0.2	44.1	±	0.3	61.3	±	0.3	0.086	±	0.001	1.009
			2015/11/13	1703	70	18.0	±	0.2	76.3	±	0.5	94.2	±	0.6	0.101	±	0.001	1.017
			2016/7/12	1945	78	16.9	±	0.2	87.1	±	0.5	104.0	±	0.6	0.103	±	0.001	1.029
		G	2016/7/12	1945	70	15.0	±	0.3	78.5	±	0.6	93.5	±	0.6	0.092	±	0.001	1.013
		03	2012/4/2	383	36	53.0	±	0.5	72.8	±	0.5	126	±	1	0.392	±	0.002	1.011
			2012/11/1	596	18	6.3	±	0.1	10.8	±	0.2	17.1	±	0.2	0.037	±	0.001	0.968
		C	2011/8/20	157	10	2.8	±	0.1	3.3	±	0.1	6.0	±	0.1	0.041	±	0.001	0.972
			2014/3/25	1105	10	0.5	±	0.0	1.2	±	0.0	1.7	±	0.1	0.003	±	0.000	1.139
		49	2012/11/1	596	26	5.3	±	0.1	9.0	±	0.1	14.3	±	0.1	0.031	±	0.000	0.983
			2015/11/13	1703	4	0.1	±	0.0	0.4	±	0.0	0.5	±	0.0	0.001	±	0.000	1.176
	Y	34	2012/11/1	596	18	1.9	±	0.1	3.3	±	0.1	5.2	±	0.1	0.011	±	0.000	0.969
		72	2012/11/1	596	16	2.0	±	0.1	3.4	±	0.1	5.4	±	0.1	0.012	±	0.000	0.964
		06	2012/11/1	596	8	0.6	±	0.0	1.1	±	0.0	1.7	±	0.1	0.004	±	0.000	0.823
		05	2013/10/4	933	30	2.3	±	0.1	5.3	±	0.1	7.6	±	0.1	0.012	±	0.000	0.969
		38	2012/4/2	383	26	3.0	±	0.1	4.2	±	0.1	7.2	±	0.1	0.022	±	0.000	0.982
			2014/3/25	1105	28	1.9	±	0.1	4.7	±	0.1	6.6	±	0.1	0.009	±	0.000	1.025
		54	2012/11/1	596	28	1.7	±	0.0	3.0	±	0.1	4.7	±	0.1	0.010	±	0.000	0.960
		61	2012/11/1	596	16	1.9	±	0.1	3.4	±	0.1	5.3	±	0.1	0.011	±	0.000	0.949
		59	2012/11/1	596	16	1.3	±	0.0	2.2	±	0.1	3.5	±	0.1	0.008	±	0.000	0.984
		36	2014/3/25	1105	10	0.3	±	0.0	0.7	±	0.0	1.0	±	0.0	0.001	±	0.000	1.059
		32	2012/11/1	596	18	3.4	±	0.1	5.6	±	0.1	9.0	±	0.1	0.020	±	0.000	1.006
		B	2011/8/20	157	16	2.3	±	0.1	2.7	±	0.1	5.0	±	0.1	0.034	±	0.001	0.958
		A	2011/8/20	157	6	0.3	±	0.0	0.3	±	0.0	0.6	±	0.0	0.004	±	0.000	0.959
			2012/11/1	596	36	4.1	±	0.1	6.9	±	0.1	11.1	±	0.1	0.024	±	0.000	0.992
	Z	07	2011/10/1	199	<5	0.3	±	0.0	0.5	±	0.0	0.8	±	0.0	0.004	±	0.000	0.825
			2011/12/3	262	<5	1.2	±	0.0	1.5	±	0.0	2.6	±	0.1	0.011	±	0.000	1.003
			2014/3/25	1105	5	0.1	±	0.0	0.4	±	0.0	0.6	±	0.0	0.001	±	0.000	0.900
			2015/11/13	1703	<5	0.1	±	0.0	0.3	±	0.0	0.4	±	0.0	0.000	±	0.000	1.040
		10	2011/10/1	199	<5	0.2	±	0.0	0.3	±	0.0	0.5	±	0.0	0.003	±	0.000	1.005
			2011/12/3	262	<5	0.2	±	0.0	0.3	±	0.0	0.6	±	0.0	0.002	±	0.000	0.833
			2014/3/25	1105	2.5	0.1	±	0.0	0.2	±	0.0	0.2	±	0.0	0.000	±	0.000	1.062
			2015/11/13	1703	<5	0.0	±	0.0	0.1	±	0.0	0.2	±	0.0	0.000	±	0.000	1.811
		H	2015/11/13	1703	<5	0.1	±	0.0	0.4	±	0.0	0.5	±	0.0	0.001	±	0.000	1.074
		69	2012/11/1	596	<5	0.3	±	0.0	0.6	±	0.0	1.0	±	0.1	0.002	±	0.000	0.907
			2014/3/25	1105	2.5	0.0	±	0.0	0.1	±	0.0	0.1	±	0.0	0.000	±	0.000	0.540
		68	2012/11/1	596	6	0.1	±	0.0	0.2	±	0.0	0.3	±	0.0	0.001	±	0.000	0.801
			2015/11/13	1703	20	0.5	±	0.1	2.3	±	0.1	2.8	±	0.1	0.003	±	0.000	0.984
		52	2012/11/1	596	14	0.7	±	0.0	1.2	±	0.0	1.9	±	0.0	0.004	±	0.000	0.993
			2014/3/25	1105	5	0.1	±	0.0	0.3	±	0.0	0.4	±	0.0	0.000	±	0.000	0.859
			2015/11/13	1703	<5	0.1	±	0.0	0.3	±	0.0	0.4	±	0.1	0.000	±	0.000	0.868
		13	2011/10/1	199	<5	0.3	±	0.0	0.4	±	0.0	0.7	±	0.0	0.004	±	0.000	0.987
			2011/12/3	262	<5	0.0	±	0.0	0.1	±	0.0	0.1	±	0.0	0.000	±	0.000	0.834
			2014/3/25	1105	2.5	0.0	±	0.0	0.1	±	0.0	0.1	±	0.0	0.000	±	0.000	0.791
			2015/11/13	1703	<5	0.0	±	0.0	0.1	±	0.0	0.1	±	0.0	0.000	±	0.000	0.914
		J	2016/7/12	1945	8	0.1	±	0.0	0.4	±	0.0	0.5	±	0.0	0.001	±	0.000	1.141
		K	2015/11/13	1703	<5	0.0	±	0.0	0.1	±	0.0	0.2	±	0.0	0.000	±	0.000	1.258
		17	2011/10/1	199	<5	0.2	±	0.0	0.3	±	0.0	0.5	±	0.0	0.003	±	0.000	0.989
			2011/12/3	262	<5	0.2	±	0.0	0.2	±	0.0	0.4	±	0.0	0.002	±	0.000	0.790
			2014/3/25	1105	2.5	0.0	±	0.0	0.1	±	0.0	0.2	±	0.0	0.000	±	0.000	0.874
			2015/11/13	1703	<5	0.0	±	0.0	0.1	±	0.0	0.1	±	0.0	0.000	±	0.000	1.283
		14	2011/10/1	199	<5	0.2	±	0.0	0.2	±	0.0	0.4	±	0.0	0.002	±	0.000	1.002
			2011/12/3	262	<5	0.2	±	0.0	0.2	±	0.0	0.3	±	0.0	0.001	±	0.000	1.309
			2014/3/25	1105	2.5	0.0	±	0.0	0.1	±	0.0	0.1	±	0.0	0.000	±	0.000	1.187
		11	2011/10/1	199	<5	0.0	±	0.0	0.2	±	0.0	0.2	±	0.0	0.001	±	0.000	0.218
			2011/12/3	262	<5	0.1	±	0.0	0.1	±	0.0	0.1	±	0.0	0.000	±	0.000	1.385
			2014/3/25	1105	2.5	0.1	±	0.0	0.2	±	0.0	0.3	±	0.0	0.000	±	0.000	0.991
			2015/11/13	1703	<5	0.0	±	0.0	0.2	±	0.0	0.2	±	0.0	0.000	±	0.000	0.957
		08	2011/10/1	199	<5	0.1	±	0.0	0.1	±	0.0	0.2	±	0.0	0.001	±	0.000	0.890
			2011/12/3	262	<5	0.2	±	0.0	0.3	±	0.0	0.5	±	0.0	0.002	±	0.000	0.999
			2014/3/25	1105	5	0.1	±	0.0	0.3	±	0.0	0.5	±	0.0	0.001	±	0.000	0.930
		09	2011/10/1	199	<5	0.2	±	0.0	0.3	±	0.0	0.5	±	0.0	0.003	±	0.000	0.920
			2014/3/25	1105	0	0.0	±	0.0	0.0	±	0.0	0.0	±	0.0	0.000	±	0.000	-
		12	2011/10/1	199	0	0.0	±	0.0	0.0	±	0.0	0.0	±	0.0	0.000	±	0.000	-
			2014/3/25	1105	2.5	0.0	±	0.0	0.1	±	0.0	0.2	±	0.0	0.000	±	0.000	0.941
		15	2011/10/1	199	<5	0.1	±	0.0	0.2	±	0.0	0.3	±	0.0	0.002	±	0.000	0.910
			2014/3/25	1105	5	0.1	±	0.0	0.2	±	0.0	0.3	±	0.0	0.000	±	0.000	0.960
		18	2011/10/1	199	<5	0.4	±	0.0	0.4	±	0.0	0.8	±	0.1	0.004	±	0.000	1.211
			2014/3/25	1105	0	0.0	±	0.0	0.0	±	0.0	0.0	±	0.0	0.000	±	0.000	-
		21	2011/10/1	199	<5	0.2	±	0.1	0.4	±	0.1	0.6	±	0.1	0.003	±	0.000	0.712
			2014/3/25	1105	0	0.0	±	0.0	0.0	±	0.0	0.0	±	0.0	0.000	±	0.000	-
		L	2015/11/13	1703	<5	0.0	±	0.0	0.2	±	0.0	0.3	±	0.0	0.000	±	0.000	1.045
		30	2011/10/1	199	<5	0.2	±	0.0	0.2	±	0.0	0.5	±	0.0	0.003	±	0.000	1.071
		24	2011/10/1	199	0	0.0	±	0.0	0.0	±	0.0	0.0	±	0.0	0.000	±	0.000	-
	W	41	2012/11/1	596	22	1.8	±	0.0	3.2	±	0.1	5.0	±	0.1	0.011	±	0.000	0.967
		43	2012/11/1	596	22	1.6	±	0.1	2.7	±	0.1	4.3	±	0.1	0.009	±	0.000	1.017
		45	2012/11/1	596	10	1.9	±	0.1	3.3	±	0.1	5.1	±	0.1	0.011	±	0.000	0.955
		57	2012/11/1	596	24	1.0	±	0.0	1.6	±	0.0	2.7	±	0.1	0.006	±	0.000	1.075
	V	19	2011/10/1	199	<5	1.0	±	0.0	1.2	±	0.1	2.2	±	0.1	0.012	±	0.000	1.024
			2011/12/3	262	<5	2.2	±	0.1	2.8	±	0.1	5.0	±	0.1	0.022	±	0.001	0.962
		22	2011/10/1	199	<5	0.5	±	0.0	0.6	±	0.0	1.1	±	0.0	0.006	±	0.000	1.003
		23	2011/10/1	199	<5	0.2	±	0.0	0.3	±	0.0	0.5	±	0.0	0.003	±	0.000	0.754
		80	2014/3/25	1105	0	0.0	±	0.0	0.0	±	0.0	0.0	±	0.0	0.000	±	0.000	-
		20	2011/10/1	199	<5	0.1	±	0.0	0.3	±	0.0	0.5	±	0.0	0.002	±	0.000	0.517
			2011/12/3	262	<5	0.3	±	0.0	0.4	±	0.0	0.6	±	0.0	0.003	±	0.000	0.995
			2014/3/25	1105	0	0.0	±	0.0	0.0	±	0.0	0.0	±	0.0	0.000	±	0.000	-

Elapsed time is from March 16, 2011 to sampling date. Contaminated layer means the thickness from the surface to deepest layer where ^134^Cs is detected. Flux was calculated by dividing the inventory by elapsed time from the FDNPP accident.

**Fig 3:**
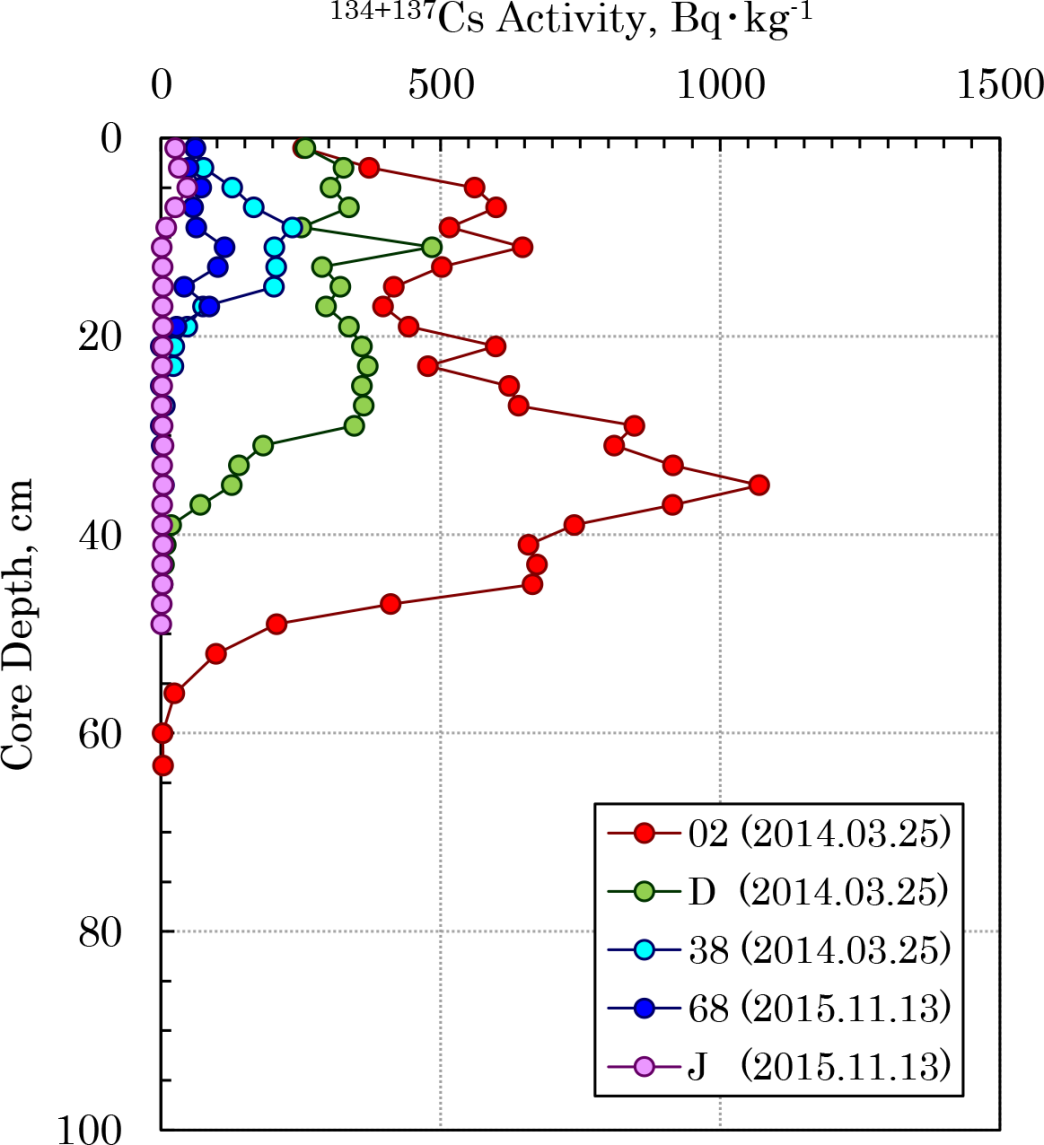
Spatial changes in the vertical distribution of ^134+137^Cs in sediment cores collected from the Old-Edogawa estuary toward the central Tokyo Bay.

The vertical distribution of radiocesium in Tokyo Bay sediment was investigated via the core samples. Fig 3 (S1 Table) shows the vertical distribution of the ^134+137^Cs activities from the Old-Edogawa mouth (Point 02) to the center of the bay (Point J) in 2014 and 2015. The highest activity of radiocesium was 1070 Bq⋅kg^-1^, detected in the 35 cm depth layer of Point 02, which is near the river mouth. However, for Point J in the center of the bay, the peak of radiocesium activity was 44 Bq⋅kg^-1^, detected in the 5 cm depth layer. This suggests that the radiocesium in the river was rapidly deposited on the sediment in the river mouth and only slightly diffused to the center of the bay.

**Fig 4:**
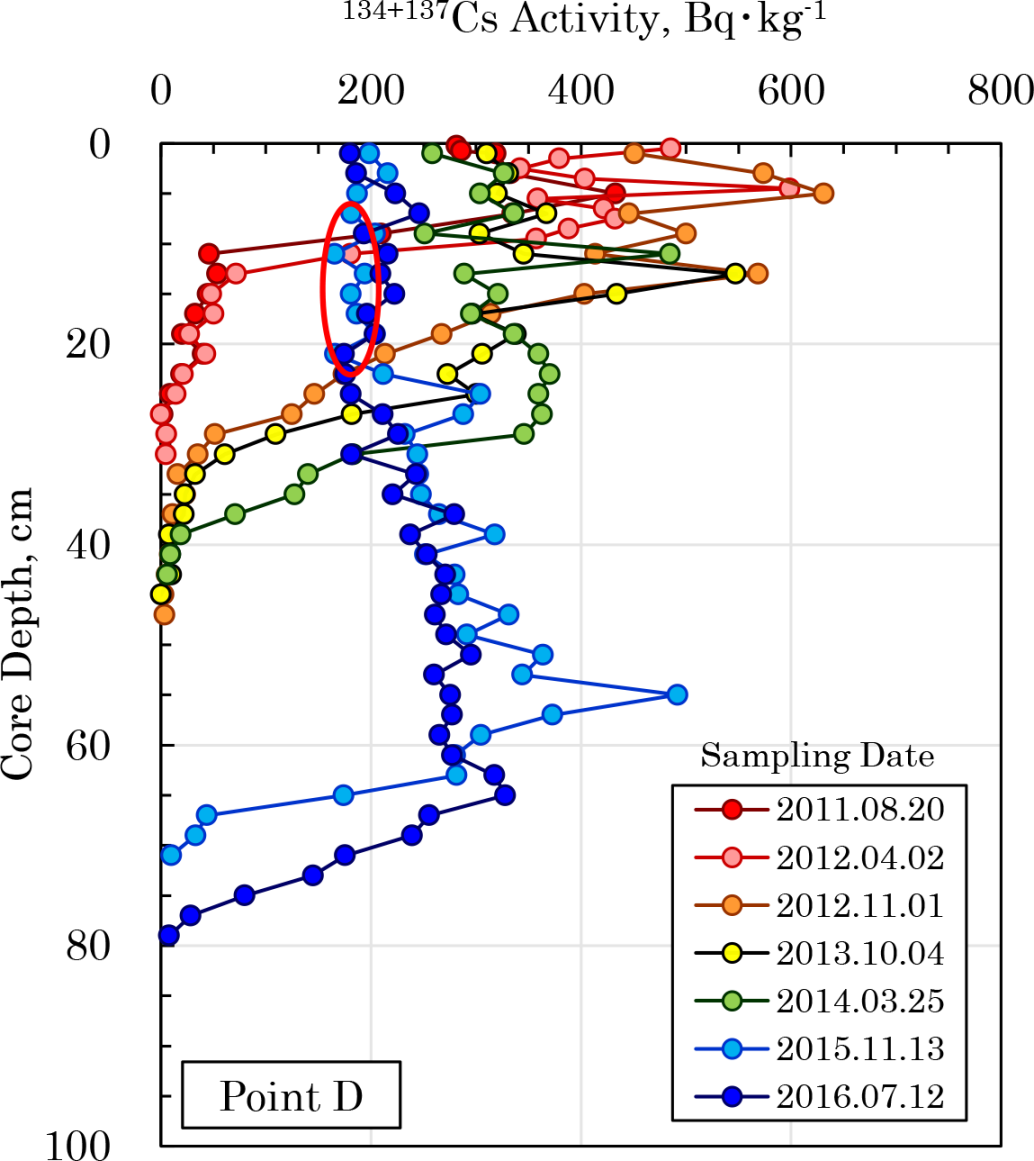
Temporal changes in the vertical distribution of ^134+137^Cs in the sediment core collected at Point D. A large amount of suspended materials flowed in owing to the Kanto-Tohoku heavy rainfall event from September 9 to 11, 2015 and was deposited in the Old-Edogawa estuary. The red circle in the core collected on November 13, 2015 shows the flood sedimentary layer that flowed in due to the flood.

The change over time in the vertical distribution of radiocesium at Point D in the Old-Edogawa estuary is shown in Fig 4 (S1 Table). The activity of radiocesium was high in the 5 cm layer in August 2011, immediately after the FDNPP accident, and it peaked at 547 Bq⋅kg^-1^ in the 13 cm layer in October 2013. Because a large amount of flood materials flowed into Tokyo Bay owing to the Kanto-Tohoku heavy rainfall event from September 9 to 11, 2015, the radiocesium was buried to a much deep layer. As shown in Fig 4 (Red circle) and S1 Fig, sediment with a relatively low deposition density was accumulated in the 8 to 22 cm layer of the Point D core collected in November 2015 after this flood. It is shown that sedimentary materials with high water content and low apparent density are deposited in the estuary area during flooding [31]. Furthermore, the radiocesium derived from the FDNPP accident was detected as approximately 350 Bq⋅kg^-1^ in the 70 cm depth layer, collected on July 2016. This value in April 2012 when correction of radioactive decay was 560 Bq⋅kg^-1^. Furthermore, It is suggested that the concentration peak of radiocesium about five years after the accident is buried while being retained in the sediment.

### Radiocesium activity in water samples around Tokyo Bay

It was assumed that radiocesium flows into Tokyo Bay through the rivers. Thus, the ^134+137^Cs activity in water from the estuaries of the major rivers that flow into Tokyo Bay was analyzed. The results are shown in Table 2. The ^134+137^Cs activity in water standardized for March 16, 2011, ranged from 4.4 to 178 mBq⋅L^-1^, and in the estuary of the Old-Edogawa, it was higher, by 20 mBq⋅L^-1^ or more. In particular, higher radiocesium activity (23 to 178 mBq⋅L^-1^) was detected in the water at the confluence of Sakagawa and Edogawa (Point S1). In the estuaries of Sumidagawa and Tamagawa, on the other hand, it was lower than 10 mBq⋅L^-1^. This conforms to the radiocesium distribution in the sediments of these points. Surface and bottom seawater was sampled from the Old-Edogawa estuary to the center of the bay, but the ratio of the ^134+137^Cs activity in the surface and bottom water was 2.05 ± 0.91 (n = 15), excluding Point J in the center of the bay, showing that the activity was approximately twofold higher in the surface water than in the bottom water. This means that the radiocesium flowed into Tokyo Bay via river water. Moreover, the radioactivity ratio of ^134^Cs/^137^Cs was 0.893 ± 0.025 (n = 51). It was thought that the water still contained 1 to 2 mBq⋅L^-1^ of ^137^Cs as background in Japan, owing to global fallout [32].

**Table 2:** Radiocesium activities in water samples collected around the Tokyo Bay water system.

Area	Point	Layer	Sampling Date	Detected, mBqL^-1^									Corrected, mBqL^-1^									Activity Ratio			
				^134^Cs			^137^Cs			^134+137^Cs			^134^Cs			^137^Cs			^134+137^Cs			(S/B)	^134^Cs/^137^Cs		
Edogawa	E1	S	2014/3/24	1.7	±	1.1	6.2	±	0.9	7.9	±	1.5	4.6	±	3.1	6.7	±	1.0	11.3	±	3.3		0.688	±	0.478
			2016/6/24	0.9	±	0.5	5.2	±	0.8	6.1	±	0.9	5.2	±	3.1	5.9	±	0.9	11.1	±	3.2		0.881	±	0.544
	E2	S	2016/6/24	1.0	±	0.4	5.8	±	0.8	6.8	±	0.9	6.0	±	2.5	6.6	±	0.9	12.5	±	2.7		0.906	±	0.402
	E3	S	2014/3/24	4.7	±	0.9	17.5	±	1.3	22.2	±	1.6	13.0	±	2.6	18.8	±	1.4	31.7	±	2.9		0.692	±	0.147
			2016/6/24	0.8	±	0.4	4.1	±	0.7	4.9	±	2.4	4.6	±	2.3	4.7	±	0.8	9.3	±	2.4		0.998	±	0.520
	E4	S	2016/6/24	0.7	±	0.5	4.9	±	0.8	5.5	±	0.9	4.1	±	3.1	5.5	±	0.9	9.5	±	3.2		0.739	±	0.568
Sakagawa	S5	S	2014/4/29	4.2	±	0.8	12.8	±	1.4	17.0	±	1.6	12.1	±	2.2	13.7	±	1.5	25.8	±	2.7		0.880	±	0.187
	S4	S	2014/4/29	12.2	±	1.2	38.9	±	1.4	51.1	±	1.8	34.9	±	3.3	41.7	±	1.5	76.6	±	3.6		0.836	±	0.085
	S3	S	2016/6/24	1.8	±	0.8	10.8	±	1.0	12.6	±	1.3	10.6	±	4.5	12.2	±	1.1	22.7	±	4.7		0.866	±	0.380
	S2	S	2016/6/24	2.7	±	0.6	17.1	±	1.2	19.8	±	1.3	16.0	±	3.3	19.3	±	1.3	35.3	±	3.6		0.829	±	0.182
	S1	S	2014/4/29	31.3	±	1.9	82.6	±	2.4	113.9	±	3.1	89.5	±	5.6	88.8	±	2.5	178.3	±	6.1		1.009	±	0.069
			2014/3/24	19.2	±	1.5	48.1	±	1.8	67.4	±	2.3	53.2	±	4.2	51.6	±	1.9	104.8	±	4.6		1.032	±	0.090
			2015/11/12	5.3	±	0.6	20.7	±	1.1	26.0	±	1.3	25.4	±	3.0	23.0	±	1.2	48.4	±	3.3		1.101	±	0.145
			2016/6/24	3.2	±	0.5	17.5	±	1.3	20.7	±	1.3	18.8	±	2.7	19.8	±	1.4	38.6	±	3.1		0.950	±	0.154
Edogawa	E5	S	2016/6/24	0.6	±	0.5	4.3	±	0.9	5.0	±	1.0	3.7	±	3.1	4.9	±	1.0	8.6	±	3.3		0.762	±	0.658
	E6	S	2014/3/24	2.2	±	0.6	5.5	±	0.7	7.7	±	0.9	6.0	±	1.5	5.9	±	0.7	11.9	±	1.7		1.010	±	0.286
			2016/6/24	2.2	±	0.6	15.4	±	1.3	17.6	±	1.5	12.9	±	3.7	17.4	±	1.5	30.3	±	4.0		0.742	±	0.225
Old- Edogawa	E7	S	2016/7/12	1.9	±	0.3	13.8	±	1.0	15.8	±	1.1	11.7	±	1.8	15.6	±	1.2	27.3	±	2.1		0.747	±	0.125
	E8	S	2016/3/24	1.4	±	0.7	8.3	±	1.0	9.8	±	1.2	7.7	±	3.7	9.4	±	1.2	17.0	±	3.9		0.817	±	0.405
			2016/7/12	1.2	±	0.5	8.9	±	0.9	10.1	±	1.1	7.5	±	3.0	10.0	±	1.1	17.5	±	3.2		0.747	±	0.309
	E9	S	2016/3/24	1.5	±	0.6	9.9	±	1.0	11.4	±	1.1	7.9	±	3.0	11.1	±	1.1	19.0	±	3.2		0.708	±	0.278
Nakagawa	N1	S	2016/7/12	1.0	±	0.4	6.7	±	0.8	7.7	±	0.9	5.7	±	2.5	7.6	±	0.9	13.3	±	2.7		0.751	±	0.343
	N2	S	2016/3/24	2.9	±	0.5	15.2	±	1.0	18.1	±	1.1	15.8	±	2.5	17.0	±	1.2	32.8	±	2.8		0.924	±	0.161
		S	2016/7/12	1.2	±	0.5	7.9	±	0.9	9.0	±	1.1	7.1	±	3.0	8.9	±	1.1	16.0	±	3.2	1.37	0.797	±	0.355
		B	2016/7/12	0.9	±	0.5	5.6	±	0.9	6.5	±	1.0	5.3	±	3.2	6.4	±	1.0	11.6	±	3.3		0.826	±	0.518
Arakawa	A1	S	2016/7/12	1.4	±	0.6	8.8	±	0.9	10.2	±	1.1	8.4	±	3.8	9.9	±	1.1	18.3	±	3.9		0.852	±	0.395
	A2	S	2016/3/24	1.8	±	0.6	9.7	±	1.1	11.5	±	1.3	9.9	±	3.4	10.9	±	1.3	20.8	±	3.6		0.915	±	0.327
		S	2016/7/12	2.3	±	0.5	12.5	±	1.1	14.7	±	1.2	13.6	±	3.3	14.1	±	1.2	27.7	±	3.5	1.31	0.967	±	0.245
		B	2016/7/12	1.5	±	0.5	10.6	±	1.1	12.1	±	1.2	9.2	±	2.9	11.9	±	1.2	21.1	±	3.2		0.771	±	0.258
	A3	S	2016/3/24	0.9	±	0.5	6.4	±	1.1	7.2	±	1.2	4.9	±	2.7	7.1	±	1.2	12.0	±	2.9		0.680	±	0.391
		S	2016/7/12	1.7	±	0.7	11.7	±	1.0	13.3	±	1.2	9.9	±	4.2	13.2	±	1.2	23.1	±	4.3	1.32	0.750	±	0.323
		B	2016/7/12	1.4	±	0.8	8.2	±	1.3	9.6	±	1.5	8.2	±	5.1	9.3	±	1.4	17.5	±	5.3		0.883	±	0.564
	A4	S	2016/7/12	2.3	±	0.6	15.4	±	0.6	17.7	±	0.9	13.9	±	3.9	17.4	±	0.7	31.3	±	3.9		0.799	±	0.224
Estuary of Arakawa and Old-Edogawa	02	S	2014/3/25	6.8	±	0.7	15.7	±	1.2	22.5	±	1.4	18.7	±	2.1	16.9	±	1.2	35.6	±	2.4	1.25	1.109	±	0.147
		B	2014/3/25	4.8	±	0.8	14.1	±	1.1	19.0	±	1.3	13.3	±	2.3	15.2	±	1.1	28.5	±	2.5		0.880	±	0.163
		S	2015/11/13	1.9	±	0.5	10.6	±	0.8	12.5	±	0.9	8.9	±	2.5	11.8	±	0.9	20.7	±	2.6	2.62	0.752	±	0.218
		B	2015/11/13	0.9	±	0.4	3.2	±	0.6	4.1	±	0.8	4.4	±	2.0	3.5	±	0.7	7.9	±	2.2		1.243	±	0.629
		S	2016/7/12	2.0	±	0.6	12.3	±	1.0	14.3	±	1.2	11.9	±	3.8	13.9	±	1.1	25.8	±	4.0	2.59	0.859	±	0.284
		B	2016/7/12	0.8	±	0.5	4.7	±	0.9	5.5	±	1.0	4.7	±	2.8	5.3	±	1.0	9.9	±	3.0		0.882	±	0.562
	64	S	2013/10/4	5.1	±	0.6	15.3	±	0.8	20.4	±	1.0	12.0	±	1.5	16.3	±	0.8	28.3	±	1.7	3.78	0.738	±	0.099
		B	2013/10/4	1.3	±	0.5	4.2	±	0.6	5.5	±	0.8	3.0	±	1.2	4.4	±	0.6	7.5	±	1.3		0.687	±	0.280
	D	S	2013/10/4	4.5	±	0.8	14.5	±	1.2	19.0	±	1.4	10.7	±	1.9	15.4	±	1.2	26.0	±	2.3	2.18	0.693	±	0.138
		B	2013/10/4	2.4	±	0.7	5.9	±	0.9	8.3	±	1.1	5.7	±	1.7	6.3	±	0.9	12.0	±	1.9		0.901	±	0.297
		S	2014/3/25	7.0	±	1.0	16.8	±	1.2	23.9	±	1.6	19.5	±	2.9	18.0	±	1.3	37.5	±	3.1	2.30	1.083	±	0.177
		B	2014/3/25	2.7	±	0.5	8.3	±	0.9	11.0	±	1.0	7.4	±	1.5	8.9	±	0.9	16.3	±	1.8		0.839	±	0.189
		S	2015/11/13	1.7	±	0.5	9.5	±	0.9	11.2	±	1.0	8.0	±	2.3	10.6	±	1.0	18.6	±	2.5	1.41	0.759	±	0.230
		B	2015/11/13	1.2	±	0.5	6.5	±	0.8	7.8	±	0.9	5.9	±	2.3	7.3	±	0.9	13.2	±	2.4		0.815	±	0.326
		S	2016/7/12	1.8	±	0.6	9.0	±	0.9	10.8	±	1.1	11.0	±	3.9	10.2	±	1.0	21.2	±	4.0	3.29	1.080	±	0.395
		B	2016/7/12	nd			5.7	±	0.9	5.7	±	0.9	-			6.4	±	1.0	6.4	±	1.0		-		
	05	S	2013/10/4	3.3	±	0.4	8.4	±	0.8	11.7	±	0.9	7.8	±	0.9	8.9	±	0.9	16.7	±	1.3	1.50	0.872	±	0.135
		B	2013/10/4	2.2	±	0.3	5.6	±	0.5	7.8	±	0.6	5.1	±	0.8	6.0	±	0.5	11.1	±	0.9		0.864	±	0.150
	38	S	2016/7/12	1.2	±	0.5	7.1	±	0.9	8.3	±	1.1	7.0	±	3.0	8.0	±	1.0	15.0	±	3.2	3.10	0.874	±	0.392
		B	2016/7/12	nd			4.3	±	0.7	4.3	±	0.7	-			4.8	±	0.7	4.8	±	0.7		-		
Center of Tokyo Bay	68	S	2014/3/25	4.9	±	0.5	13.1	±	1.0	18.0	±	1.1	13.6	±	1.3	14.0	±	1.1	27.6	±	1.7	2.71	0.972	±	0.120
		B	2014/3/25	2.0	±	0.3	4.3	±	0.6	6.3	±	0.7	5.6	±	0.8	4.6	±	0.7	10.2	±	1.1		1.210	±	0.252
	J	S	2015/11/14	0.5	±	0.4	1.9	±	0.5	2.4	±	0.7	2.3	±	2.2	2.1	±	0.6	4.4	±	2.2	0.66	1.075	±	1.061
		B	2015/11/14	0.7	±	0.5	2.8	±	0.6	3.5	±	0.8	3.5	±	2.4	3.1	±	0.7	6.6	±	2.5		1.122	±	0.795
Sumidagawa	M1	S	2016/3/24	1.4	±	0.6	6.5	±	0.8	7.9	±	1.0	7.9	±	3.4	7.3	±	0.9	15.1	±	3.6		1.083	±	0.495
	M2		2016/3/24	0.4	±	0.7	3.2	±	0.7	3.6	±	0.9	2.2	±	3.7	3.6	±	0.7	5.8	±	3.7		0.627	±	1.037
Tamagawa	T1	S	2016/3/24	0.3	±	0.4	2.6	±	0.7	2.9	±	0.8	1.6	±	2.0	2.9	±	0.8	4.5	±	2.2		0.544	±	0.702
	T2		2016/3/24	0.5	±	0.6	3.3	±	0.7	3.8	±	0.9	2.9	±	3.2	3.7	±	0.8	6.6	±	3.3		0.784	±	0.877
Tsurumigawa	Z1	S	2016/3/24	0.6	±	0.6	3.6	±	0.9	4.2	±	1.1	3.4	±	3.4	4.0	±	1.0	7.4	±	3.5		0.851	±	0.871
	Z2		2016/3/24	0.6	±	0.4	4.3	±	0.7	4.9	±	0.8	3.4	±	2.1	4.8	±	0.8	8.2	±	2.3		0.720	±	0.461

The corrected value was radioactive decay corrected based on the value of March 16, 2011. The ^134^Cs/^137^Cs activity ratio was calculated as a weighted average value without the data for counting error more than 30%. S: Surface water, B: Bottom water above 1 m from seabed. Activity ratio of the surface layer to the bottom layer (S/B) was calculated with the radioactive decay corrected values.

### Spatiotemporal distribution of radiocesium in the river sediment of Old-Edogawa, Edogawa, and Sakagawa

In the Sakagawa (Fig 2), which converges with the middle Edogawa, the weighted average ^134+137^Cs activity was 3630 ± 11 Bq⋅kg^-1^ (n = 11), whereas in Edogawa, it was 170 ± 2 Bq⋅kg^-1^ (n = 20) upstream from Sakagawa and 473 ± 3 Bq⋅kg^-1^ (n = 14) downstream from Sakagawa. As the results of the aircraft monitoring [2] in Figs 1 and 2 clearly show, the forested zone in Gunma Prefecture upstream on Tonegawa River was also contaminated with radiocesium at 60 to 300 kBq⋅m^-2^. However, upstream in Edogawa after it diverges from Tonegawa, the ^134+137^Cs activity was low in both the sediment and water at Point E4. Therefore, it considered that the radiocesium-contaminated area in upstream Tonegawa is not an important radiocesium source supplying Tokyo Bay.

The Matsudo Weir is installed about 100 m downstream from Point S1 and is normally closed to adjust the flow rate of the Sakagawa water. This means that contaminated suspended materials flowing down from the Sakagawa catchment basin is deposited at the point where the flow velocity is low. However, this weir was opened during the Kanto-Tohoku heavy rainfall event from September 9 to 11, 2015 [33,34], and the sediment deposited upstream of the weir flowed out into the confluence with Edogawa.

**Fig 5:**
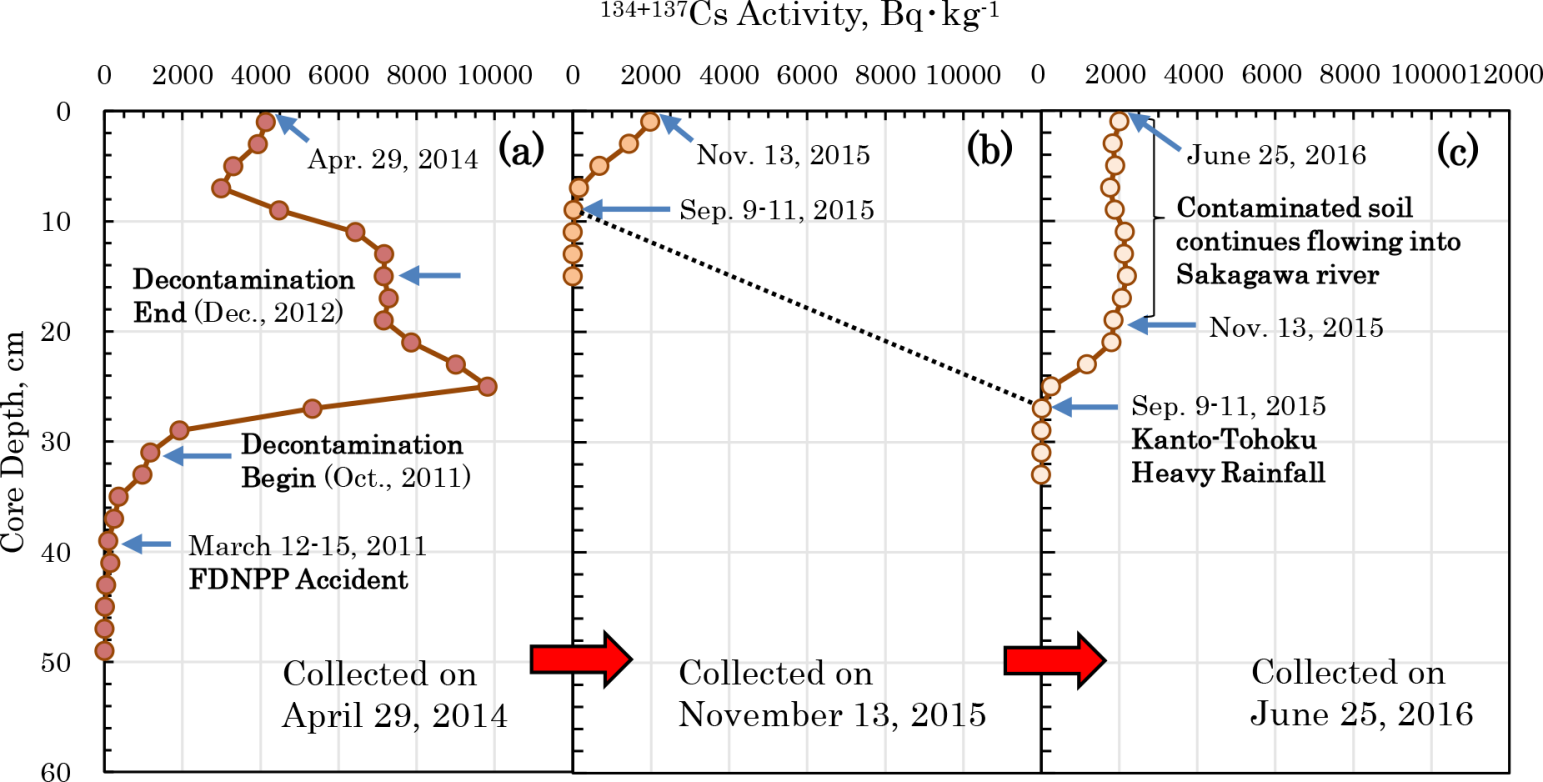
Temporal changes in the vertical distribution of ^134+137^Cs in the sediment core at Point S1. The core samples were collected on (a) April 29, 2014; (b) November 13, 2015; and (c) June 25, 2016. When the weir was opened because of the Kanto-Tohoku heavy rainfall event. A large proportion of the contaminated sediment deposited at Point S1 flowed out. The period of decontamination work in Kashiwa City in the Sakagawa catchment area as shown in “Begin” and “End” of Fig 5A [35].

The core sampled on April 29, 2014 (Fig 5A, S3 Table) shows a record of the vertical distribution of radiocesium from before the FDNPP accident. If it is presumed that the 38 to 40 cm layer in which ^134^Cs was detected is the layer was deposited immediately after the accident, the rate of deposition of this sediment is 1.0 cm⋅month^-1^. Thus, the sediment layer deposited in October 2011, when decontamination work started in Kashiwa City [35], which is in the catchment basin of Sakagawa, is the sediment layer from 32 to 34 cm. This decontamination work was completed in December 2012, which corresponds to the layer from 14 to 16 cm. For this reason, the vertical distribution of ^134+137^Cs, shown in Fig 5A, presumably is sediment with a record of contaminated soil from the high contamination zone accompanied by a discharge of contaminated sludge caused by the decontamination work in Kashiwa City. Some of the decontamination wastewater that does not receive treatment flows into Sakagawa, and it is highly likely that this contaminated sludge was deposited at Point S1. In a core sampled at the same point on November 13, 2015, three months after the Kanto-Tohoku heavy rainfall event, the high contamination sediment layer had disappeared, but new contaminated sediment had been deposited (Fig 5B). The sedimentation rate at Point S1 after the flood was 2.2 cm⋅month^-1^, suggesting that the sedimentation environment had changed under the effects of the flood. Another core sampled in July 2016 showed a sedimentation rate of 2.2 cm⋅month^-1^, suggesting that, similarly to the core after the flood (Fig 5B), the ^134+137^Cs activity was a constant value of about 2,000 Bq⋅kg^-1^ (Fig 5C). This implies that contaminated soil has been flowing constantly into Sakagawa from the high contamination zone around Kashiwa, and thus is a supply source of radiocesium that has accumulated in the Old-Edogawa estuary in Tokyo Bay.

## Discussion

### Importance of Sakagawa River as a radiocesium source supplying Tokyo Bay

As shown in Fig 2, the radiocesium activity of the sediments in the Sakagawa, Edogawa and Old-Edogawa rivers is higher than that in the Edogawa upstream. Furthermore, it is suggested from Fig 5 that contaminated soils flowing in Sakagawa from the high contaminated zone around Kashiwa City are being transported to Tokyo Bay through these rivers. In other words, the origin of radiocesium contamination in Tokyo Bay is polluted soil in the high contaminated zone, and it can be thought that the Edogawa water system plays an important role in its transportation.

**Fig 6:**
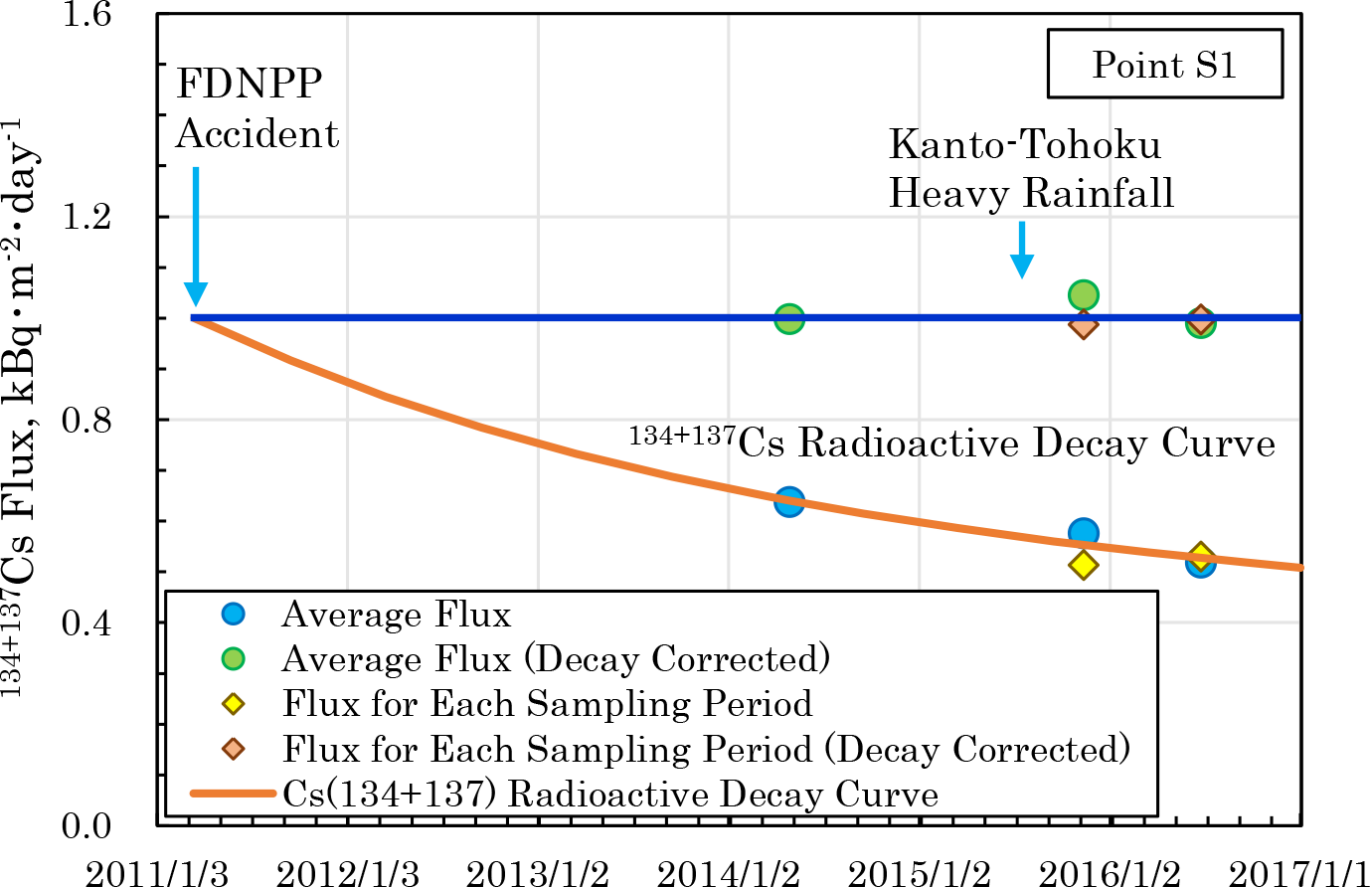
Temporal changes in the estimated flux of ^134+137^Cs to the sediment at Point S1. The radioactive decay curve of ^134+137^Cs was calculated assuming that the activities of ^134^Cs and ^137^Cs were equal immediately after the FDNPP accident [28–30].

Fig 6 (S7 Table) shows the flux of the ^134+137^Cs under the sediments at Point S1 calculated from Fig 5 (S3 Table). The estimated flux immediately after the accident was about 1.0 kBq⋅m^-2^⋅day^-1^ and had decreased to about 0.5 kBq⋅m^-2^⋅day^-1^ by July 2016. Fig 6 also shows the flux estimated for each the sampling period of each sediment core, and the flux decay corrected based on the value of March 16, 2011. For both, the radioactive decay curve of ^134+137^Cs conforms closely, and from this fact as well, it was assumed that a constant supply of radiocesium currently continues at Point S1.

### Contamination via global fallout of ^137^Cs in Tokyo Bay sediment before the FDNPP accident

Compared with the water samples, the radiocesium activity in the present soil and sediment samples before the FDNPP accident is extremely low; therefore, we did not consider the background in this study.

Global fallout via atmospheric nuclear testing contributed ^137^Cs to the environment before the FDNPP accident. Precipitation of the global fallout ^137^Cs from the atmosphere was 6.56 kBq⋅m^-2^ from 1954 to 1985 (before the Chernobyl accident), in Chiba City, located in east of Tokyo Bay (S8 Table) [36]. The inventory of the global fallout ^137^Cs in the sediment of central Tokyo Bay was presumed from 0.37 to 0.51 kBq⋅m^-2^ [37]. However, it is possible these values are underestimates, as the cores were too short. The global fallout ^137^Cs in the initial stage probably was not measured. On the other hand, we also found a record of the global fallout ^137^Cs in the core collected from Points 36 and J in the center of Tokyo Bay. The inventories were 0.80 and 0.71 kBq⋅m^-2^, respectively, for Points 36 and J (S8 Table). From our data, it was estimated that 11 to 12% of the global fallout ^137^Cs precipitation observed in Chiba City from 1954 to 1985 was accumulated in the Tokyo Bay sediment. However, as shown in Table 2, it is presumed from the ^134^Cs/^137^Cs activity ratio that ^137^Cs due to the global fallout and the fallout from the Chernobyl accident is significant in river water and seawater in the Tokyo Bay water system.

### Inventory and flux of radiocesium owing to the FDNPP accident in the sediment of Point D in the Old-Edogawa estuary

**Fig 7:**
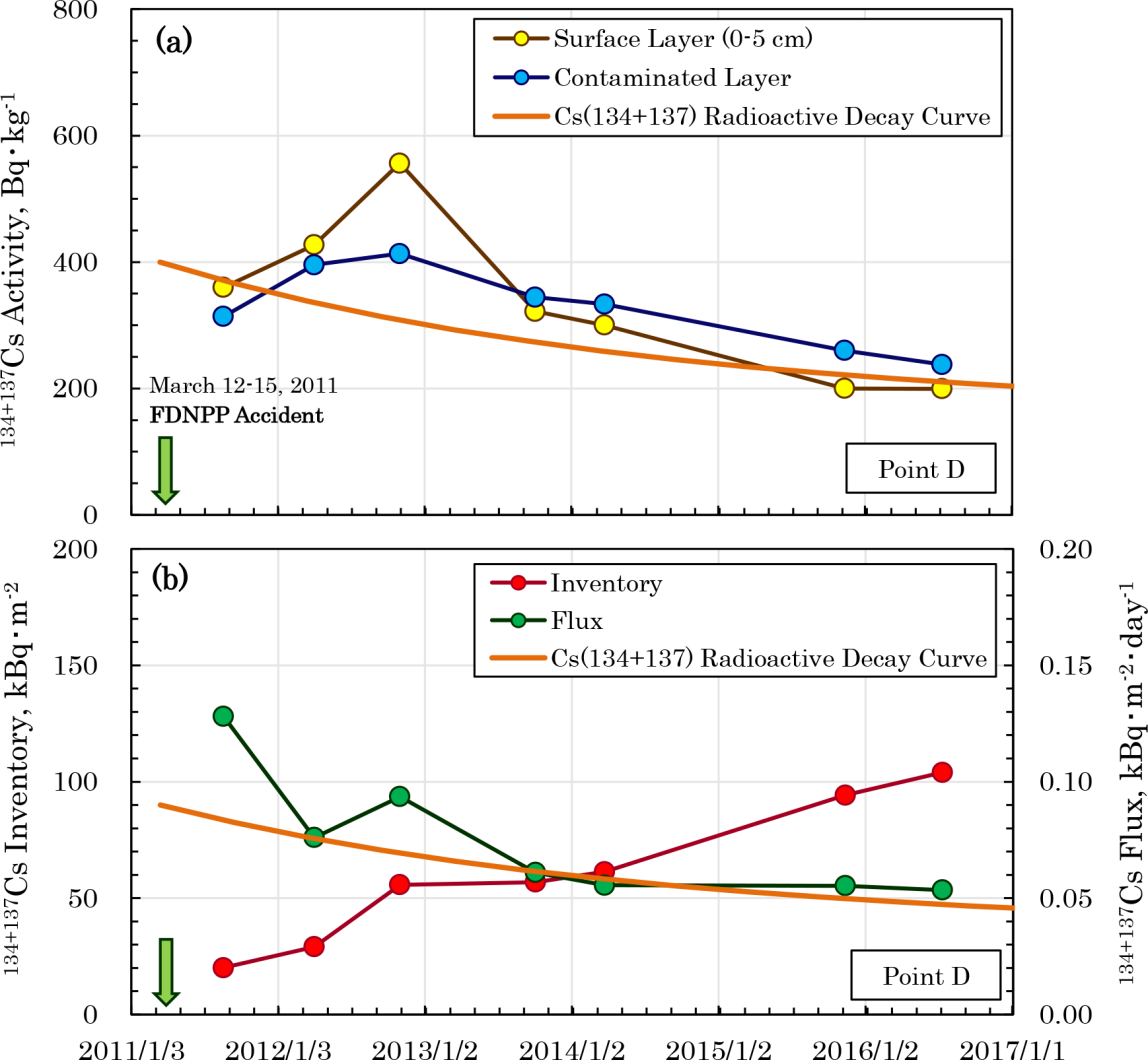
**Temporal changes in activity (a) and inventory and flux (b) of** ^**134+137**^**Cs at Point D.** Contaminated layer is defined as the layer including the detectable ^134^Cs. The ^134+137^Cs activity in the contaminated layer is estimated by the vertical distribution in the layer. Flux was calculated by dividing the inventory by elapsed time since the FDNPP accident.

Based on the vertical distribution of radiocesium in the core sediment deposits at Point D, as shown in Fig 4, the changes over time in the activity, inventory, and flux of the ^134+137^Cs in the sediment were analyzed. The results are shown in Fig 7 (S9 Table). The radioactive decay curve of the ^134+137^Cs activity leads to the hypothesis that the ^134^Cs/^137^Cs activity ratio emitted during the accident was 1.0 [28–30]. The ^134+137^Cs activity of surface sediment presumably reflects the level of contamination of the catchment basin, which is the supply source of radiocesium. Fig 7A shows the average radioactivity of ^134+137^Cs in the sediment layers contaminated by radiocesium and in the top 5 cm of the sediments. In both the surface and in the contaminated layers, the maximum values appeared at the end of 2012, about two years after the accident. Afterwards, the ^134+137^Cs activity of sediments decreased until its value matched the activity anticipated from the radioactive decay curve. Fig 7B shows the inventory and change over time of ^134+137^Cs in sediments and the flux estimated from the inventory. The inventory immediately after the accident was about 20 kBq⋅m^-2^, but five years later in 2016, it had increased to about 100 kBq⋅m^-2^. Theoretically, by that time, the ^134+137^Cs activity should have decayed to 53% of its value immediately after the accident, yet the inventory had greatly increased. The flux was 0.13 kBq⋅m^-2^⋅day^-1^ immediately after the accident, but in 2016, it had decreased to 0.053 kBq⋅m^-2^⋅day^-1^ (19 kBq⋅m^-2^⋅yr^-1^). Beginning in 2014, when it is assumed that the inflow of radiocesium to Point D had become constant, the flux also conformed almost exactly to the values anticipated, based on the radioactive decay of ^134+137^Cs. This suggests that, even now, radiocesium continues to constantly flow into Tokyo Bay.

### Balance of the radiocesium flowing into Tokyo Bay from the Edogawa watershed

Based on the analytical results, the balance of radiocesium in the Edogawa water system and in Tokyo Bay sediment are shown in Table 3 (S2 Fig). Under the activity value standardized to that of March 16, 2011, the quantity of ^134+137^Cs precipitated in the catchment basin of the Edogawa water system was 8.33 TBq per the results of aircraft monitoring [2]. Judging from the results of the analysis of the core samples, the average inventory of ^134+137^Cs in Area X, which has an area of 10 km^2^ and is located about 8 km southeast of Chiyoda-Ward in central Tokyo, was 131 kBq⋅m^-2^ (n = 10), and the total inventory of ^134+137^Cs was 1.31 TBq. In July 2016, about 70% of the radiocesium deposited in the study area of Tokyo Bay accumulated in Area X. Similarly, the average inventory in Area Y, which is 40 km^2^ in area, was 5.52 kBq⋅m^-2^ (n = 11), and the total inventory was 0.22 TBq. Assuming that all the radiocesium deposited in Area X was supplied from the Edogawa water system, about 16% of radiocesium precipitated in the catchment basin during the five years following the accident had moved to Area X. This value is larger than the 11 to 12% global fallout measured in the center of Tokyo Bay, but because Area X is located in the estuary, it is reasonable that it has a relatively large value. Furthermore, the contaminated particles that flowed out during the decontamination work in the highly contaminated zone also may have caused this value to be higher. The average flux in sediment of Area X for 5.4 years (from the FDNPP accident to July 2016) was 0.067 kBq⋅m^-2^⋅day^-1^ (24.5 kBq⋅m^-2^⋅yr^-1^), so 0.245 TBq of radiocesium continues to flow into Area X every year. This value is consistent with the value for July 2016 of the flux estimated at Point D, off the coast of the river mouth, as shown in Fig 7. The average radiocesium flux of Area X in July 2016 from the FDNPP accident is about 15 times the maximum value of 1.59 kBq⋅m^-2^⋅yr^-1^ of the global fallout ^137^Cs, found in 1963 in Chiba City [36].

**Table 3:** Balance of the radiocesium precipitated in the Edogawa watershed.

Region			Area^a^	^134+137^Cs Activity			Ratio for Total Amount
				Inventory	Each Amount	Total Amount	
			km^2^	kBq⋅m^-2^	TBq	TBq	%
*Source*							
	Edogawa Upstream	A	50	10	0.50	8.33	
	Kashiwa City	B	20	80	1.60		
	Edogawa Midstream	C	65	45	2.93		
	Edogawa Downstream	D	80	20	1.60		
	Nakagawa	E	85	20	1.70		
*Sink*							
	Old-Edogawa Estuary	X	10	131	1.31	1.53	15.7
	Offshore from Area X	Y^b^	40	5.52	0.22		2.6
	Central Bay	Z^b^, V^b^	330	0.73	0.24	0.24	2.9
	Sumidagawa Estuary	W^b^	20	5.55	0.11	0.11	1.3

The ^134+137^Cs activities are shown the values at July 2016 as the radioactive decay corrected on March 16, 2011. The inventory of source was estimated from the result of the MEXT aircraft monitoring [2]. The inventory of sink was calculated from the analytical values of core sediments.

^a^Areas shown in S2 Fig.

^b^It is thought that the radiocesium flowing in from other than the Edogawa watershed also accumulates.

### Estimation of the sedimentation process of radiocesium in the Edogawa water system and Tokyo Bay

**Fig 8:**
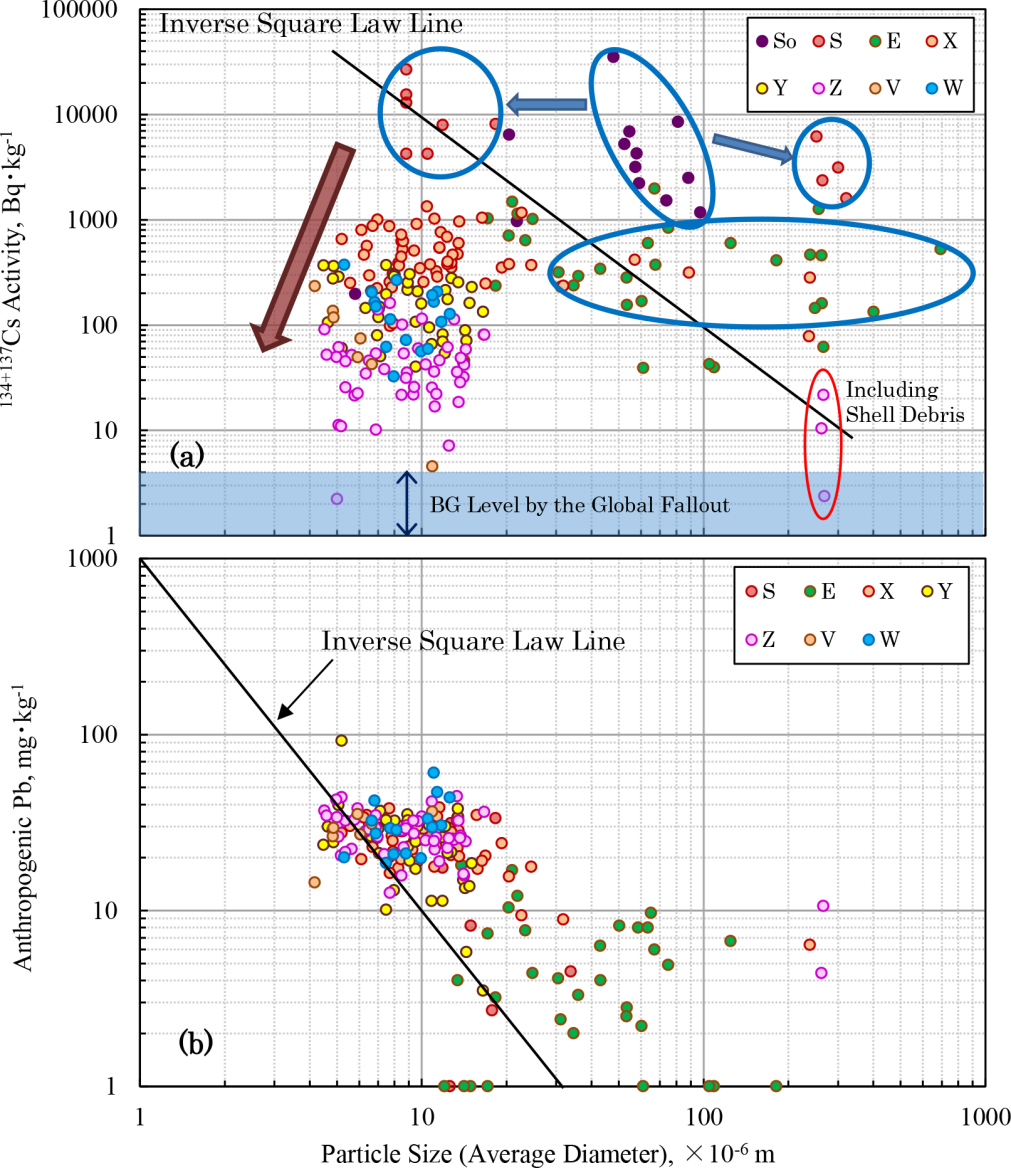
Relationship between the ^134+137^Cs activity and the grain size of surface sediments in the Tokyo Bay water system. So: Soil in the Sakagawa catchment area, S: Sakagawa river, E: Edogawa and Old-Edogawa rivers, X: Old-Edogawa estuary, Y: Off the Old-Edogawa estuary, Z: Center of Tokyo Bay, V: Tamagawa estuary, W: Sumidagawa estuary. If the radiocesium is adsorbed on the suspended materials in water owing to the grain size effect [56–58], the relationship should obey the inverse square law. The vertical arrow in the Fig 8A indicates the range of the radiocesium background referred from the JCG report [23].

Model calculation was conducted and the process of the movement of radiocesium from the land to the aquatic system was simulated based on values observed in various environments [38–41]. Our study has shown that radiocesium precipitated in the northeastern part of the greater Tokyo region was accumulated through the rivers into the estuaries of deep in Tokyo Bay without diffusing to the center of the bay (Figs 2, 3 and 8A, S6 Table). Changes in the particle size distribution of sediment from Kashiwa City to the center of Tokyo Bay shown in S3 Fig support the existence of radioactive cesium deposition process in this area. That is, Sakagawa Point S5 has contaminated soil flowing in from Kashiwa City. Since the flow is fast, large particles (200–1000 μm) are selectively deposited. In the Matsudo Weir of Point S1, the flow velocity of the water slows down as that fine particles (0.3–0.5 μm, red circle in S3 Fig) are also deposited. The fine particles are not found in sediments at Points 02 and D of the estuary where seawater and river water mix. This suggests that coagulated deposition of colloidal particles by salting out did not occur. Since the average particle size of the sediment decreases from the estuary towards the offshore, it is shown that sedimentary substances flowing from the river are accumulating first from large particles as they diffuse through the sea water.

It is well known that cesium cations is inserted between layers of 2:1:1 type clay minerals like vermiculite and strongly absorbs by ion exchange [42–47]. Therefore, radiocesium precipitated from atmosphere on ground surfaces is absorbed and held by clay minerals in the soil. Recently, it has been confirmed directly that radiocesium released from the FDNPP is adsorbed by the clay mineral using a new technic such as EXAFS and NMR [48–50]. Moreover, fine particles with a particle size of 10 μm or less containing radiocesium at high activity are found in various places of eastern Japan [51–55], but we believe that washout due to rainfall plays an important role in precipitation on the ground even if radiocesium is scattered in the atmosphere as gaseous or fine particles. It can be hypothesized that in the greater Tokyo region, radiocesium precipitated on ground surfaces was absorbed by the soil and transported to the Tokyo Bay estuary by such a mechanism, underwent coagulating sedimentation via the salting out effect as it mixed with seawater [56,57], and then accumulated in the estuary sediment. However, such a mechanism cannot explain the difference in the sedimentation processes of radiocesium and that of heavy metals in Tokyo Bay (Fig 8, S6 Table, S3 Fig).

Assuming that the background lead concentration in the Tokyo Bay sediment older than 1800s is about 10 mg⋅kg^-1^ [59], the anthropogenic lead concentration of the sediment was estimated. As shown in Fig 8B, a clear grain size effect between the concentrations of lead and the average particle size of the surface sediments in the Tokyo Bay water system has been confirmed. This indicates that the lead is selectively accumulated in sediments in the center of Tokyo Bay, where the average particle size is relatively small. This phenomenon occurs when anthropogenic lead absorbs onto the surface of suspended particles in water. But the slope is less than two. As many rivers flow into Tokyo Bay, this may be due to the dilution effect of suspended particles with low anthropogenic lead contamination. Moreover, as the particle size increases, it deviates slightly from the inverse square law. This is probably due to the relatively higher lead concentration of constituent components of sediments than anthropogenic lead in large particles. Such a tendency was also observed with zinc and mercury (S6 Table, S4 Fig). If it can be assumed that the deposited particle are spherical, the radiocesium activity should follow the inverse square law for the sediment particles [56–58]. However, a negative correlation between them is not observed.

As shown in Fig 8, the average particle size in the soil of the high contamination zone was 20 to 100 μm and the radiocesium activity was in the range of 1000 to 35000 Bq⋅kg^-1^. It is believed that this soil in the high contamination zone is the source of radiocesium contamination in Tokyo Bay. When it flows into Sakagawa, the average particle size is separated into 8 to 20 μm (4000 to 35000 Bq⋅kg^-1^) and 250 to 350 μm (1500 to 6000 Bq⋅kg^-1^) size classes. The larger particles settle in Sakagawa, and the smaller particles move to the confluence with Edogawa. From Sakagawa to Tokyo Bay, there seems to be a positive correlation in the process between the particle size and the radiocesium activity as opposed to the grain size effect. This suggests that the radiocesium is not adsorbed to suspended particles in water during transportation from Sakagawa to Tokyo Bay. The low radiocesium activity in Area Z in the central part of Tokyo Bay, where the sediment particles are smallest, is thought to be due to the dilution effect observed, the same as for the heavy metals. The relationship between radiocesium activity and particle size in Tokyo Bay is much different from the relationship observed in the estuary area of Abukuma River, which flows through the most contaminated zone owing to the FDNPP accident [60–62]. This is probably because the Abukuma River estuary faces the Pacific Ocean, hence particle size fractionation of the contaminated suspended particles due to tidal currents and waves is occurring. On the other hand, because Tokyo Bay is closed, it is not affected by the ocean waves. Therefore, the large particles contaminated with radiocesium are selectively deposited in the river mouth as the flow rate decreases, and small particles are transported to the center of the bay, where they settle. The distribution in Fig 8A (S3 Fig) can be explained by such a mechanism. When radiocesium adsorbed onto fine clay minerals flows from the river into Tokyo Bay, there is a possibility of coagulation and precipitation owing to the salting out effect of seawater [42,56,57], but from our results regarding this period, no such effect was found.

## Conclusions

Changes in the radiocesium contamination of the Tokyo Bay sediment surrounded by the Tokyo metropolitan area were discussed for five years after the FDNPP accident. Most of radiocesium flowed from the high contaminated zone in the northeastern part of the Tokyo metropolitan area through the river into the Old-Edogawa estuary inner part of Tokyo Bay and was accumulated without diffusing to the center of the bay. The radioactivity increased from immediately after the accident and decreased following the theoretical radioactive decay after showing the maximum value of 547 Bq⋅kg^-1^ at end of 2012. The average inventory of radiocesium in the Old-Edogawa estuary increased to 104 kBq⋅m^-2^ in July 2016. At that time about 70% of the radiocesium deposited in the study area of Tokyo Bay had accumulated in the Old-Edogawa sediment. On the other hand, the inventory of radiocesium in the central bay was 0.46 kBq⋅m^-2^.

Radiocesium may be transported to Tokyo Bay as contaminated soil particles through rivers such as Sakagawa, Edogawa, and Old-Edogawa from the high contaminated zone in the northeastern part of the Tokyo metropolitan area. Observation results suggested that the river plays an important role in transporting radiocesium from pollution sources. Furthermore, it is considered that the radiocesium precipitated from the atmosphere was adsorbed onto soil particles. The contaminated particles flow out into the river, but they selectively deposited and accumulated in the estuary of Tokyo Bay where the flow velocity of the river water decreases. Therefore, it was presumed that it did not diffusing to the center of the bay.

It is important to recognize that, as the present study has indicated, radiocesium from the FDNPP accident which was deposited in distant watersheds is still flowing into Tokyo Bay, and the consequences are not fully understood. Continued and careful long-term monitoring of these environmental radionuclides is warranted. In the other words, Tokyo Bay plays the role of a sink for radioactive contaminants discharged in the greater Tokyo region.

## Supporting information

S1 Fig Evidence that flood sediment deposited at Point D. Coastal flood sediments have high water content and small particle size, so their apparent density is small. The cumulative mass of the 8 to 22 cm layer of the core collected in November 2015 immediately after the Kanto-Tohoku heavy rainfall event is clearly lower than that of the other cores. It can be thought that this is a trace of the flood sedimentary layer [31].
(DOCX)

S2 Fig Classification region map for radiocesium balance estimation in Tokyo Bay and Edogawa watershed. The area surrounded by the red line shows the Edogawa river catchment basin. Area E indicates the Nakagawa river catchment basin of a branch of Edogawa.
(PPTX)

S3 Fig Change in the particle size distribution of surface sediments in the Tokyo Bay water system. The green arrow indicates the average particle diameter.
(PPTX)

S4 Fig Relationship between heavy metal and particle size of surface sediments in the Tokyo Bay water system. S: Sakagawa river, E: Edogawa and Old-Edogawa rivers, X: Old-Edogawa estuary, Y: Off the Old-Edogawa estuary, Z: Center of Tokyo Bay, V: Tamagawa estuary, W: Sumidagawa estuary.
(PPTX)

S5 Fig Photographs of the sampling location and sampling sediment. (a) Viewing the Tokyo metropolitan from Area X. (b) Core sampling by a diver. (c)-(e) Sediment core in seabed and the collected core sample. (f), (g) Surface sediment collected by an Ekman-Birge sampler.
(PPTX)

S1 Table Radiocesium activity and particle size in the sediment samples collected from Tokyo Bay. Corrected values were corrected for radioactive decay on March 16, 2011.
(XLSX)

S2 Table Radiocesium activity and particle size in the sediment samples collected from Edogawa River. Corrected values were corrected for radioactive decay on March 16, 2011.
(XLSX)

S3 Table Radiocesium activity, inventory, and particle size in the sediment samples collected from Sakagawa River. Corrected values were corrected for radioactive decay on March 16, 2011.
(XLSX)

S4 Table Radiocesium activity, anthropogenic heavy metal concentration, and particle size in the soil collected around Kashiwa City. Corrected values were corrected for radioactive decay on March 16, 2011. The background concentration of heavy metal was assumed to be Zn 100 mg⋅kg^-1^, Hg 40 μg⋅kg^-1^, and Pb 10 mg⋅kg^-1^.
(XLSX)

S5 Table Geographic coordinates of sampling points. Latitude and longitude of land sampling points were not measured.
(XLSX)

S6 Table Radiocesium activity, anthropogenic heavy metal concentration, and particle size in the surface sediment collected from the Tokyo Bay water system. a: X (Old-Edogawa estuary), Y (Off the Old-Edogawa estuary), Z (Center of Tokyo Bay), V (Tamagawa estuary), W (Sumidagawa estuary). b: Value on sampling date. c: Corrected values were corrected for radioactive decay on March 16, 2011. d: Weighted average value of the decay corrected radiocesium activity for multiple samples from the same site. e: Ratio for the decay corrected value. f: Value for samples whose counting error of the decay corrected total radiocesium activity was within 5%. The background concentration of heavy metal was assumed to be Zn 100 mg⋅kg^-1^, Hg 40 μg⋅kg^-1^, and Pb 10 mg⋅kg^-1^.
(XLSX)

S7 Table Estimated radiocesium flux at Point S1 in Sakagawa. a: Corrected values were corrected for radioactive decay on March 16, 2011. The values in parentheses are the detected values for the sampling date. b: Contaminated sediment flowed out owing to the Kanto-Tohoku heavy rainfall event of September 9 to 11, 2015. c: Sampling interval.
(XLSX)

S8 Table Estimated inventory of the global fallout ^137^Cs recorded in the core sediment at Points 36 and J in Tokyo Bay and the annual precipitation of global fallout ^137^Cs at Chiba City. Sedimentary age of the sediment was estimated by the ^210^Pb method. a: The activity was corrected for radioactive decay in the sedimentary age. b: The value measured at Chiba City by the NIRS and published by the JCAC [36].
(XLSX)

S9 Table Flux and inventory of radiocesium in the sediment collected at Point D of the Old-Edogawa estuary. a: Including the flood-deposited layer of 8 to 22 cm (average activity: 170 Bq⋅kg^-1^). b: Including the flood-deposited layer in the core. c: Weighted average of ratio of ^134^Cs to ^137^Cs in the sediment of each layer.
(XLSX)
