# Supplementary material for: Spatiotemporal distribution and fluctuation of radiocesium in Tokyo Bay in the five years following the Fukushima Daiichi Nuclear Power Plant (FDNPP) accident
Source: PLoS One. 2018 Mar 1;13(3):e0193414. doi: 10.1371/journal.pone.0193414 (PMC5832246; doi:10.1371/journal.pone.0193414)
Supplement: S1 Fig — Coastal flood sediments have high water content and small particle size, so their apparent density is small. The cumulative mass of the 8 to 22 cm layer of the core collected in November 2015 immediately after the Kanto-Tohoku heavy rainfall event is clearly lower than that of the other cores. It can be thought that this is a trace of the flood sedimentary layer [31]. (DOCX) [file pone.0193414.s001.docx]

**S1 Fig. Evidence that flood sediment deposited at Point D.** Coastal flood sediments have high water content and small particle size, so their apparent density is small. The cumulative mass of 8 to 22 cm layer of the core collected in November 2015 immediately after the Kanto-Tohoku heavy rainfall event is clearly lower than that of the other cores. It can be thought that this is a trace of the flood sedimentary layer [31].
