# Supplementary material for: Spatiotemporal distribution and fluctuation of radiocesium in Tokyo Bay in the five years following the Fukushima Daiichi Nuclear Power Plant (FDNPP) accident
Source: PLoS One. 2018 Mar 1;13(3):e0193414. doi: 10.1371/journal.pone.0193414 (PMC5832246; doi:10.1371/journal.pone.0193414)
Supplement: S2 Fig — The area surrounded by the red line shows the Edogawa river catchment basin. Area E indicates the Nakagawa river catchment basin of a branch of Edogawa. (PPTX) [file pone.0193414.s002.pptx]

## Slide 1
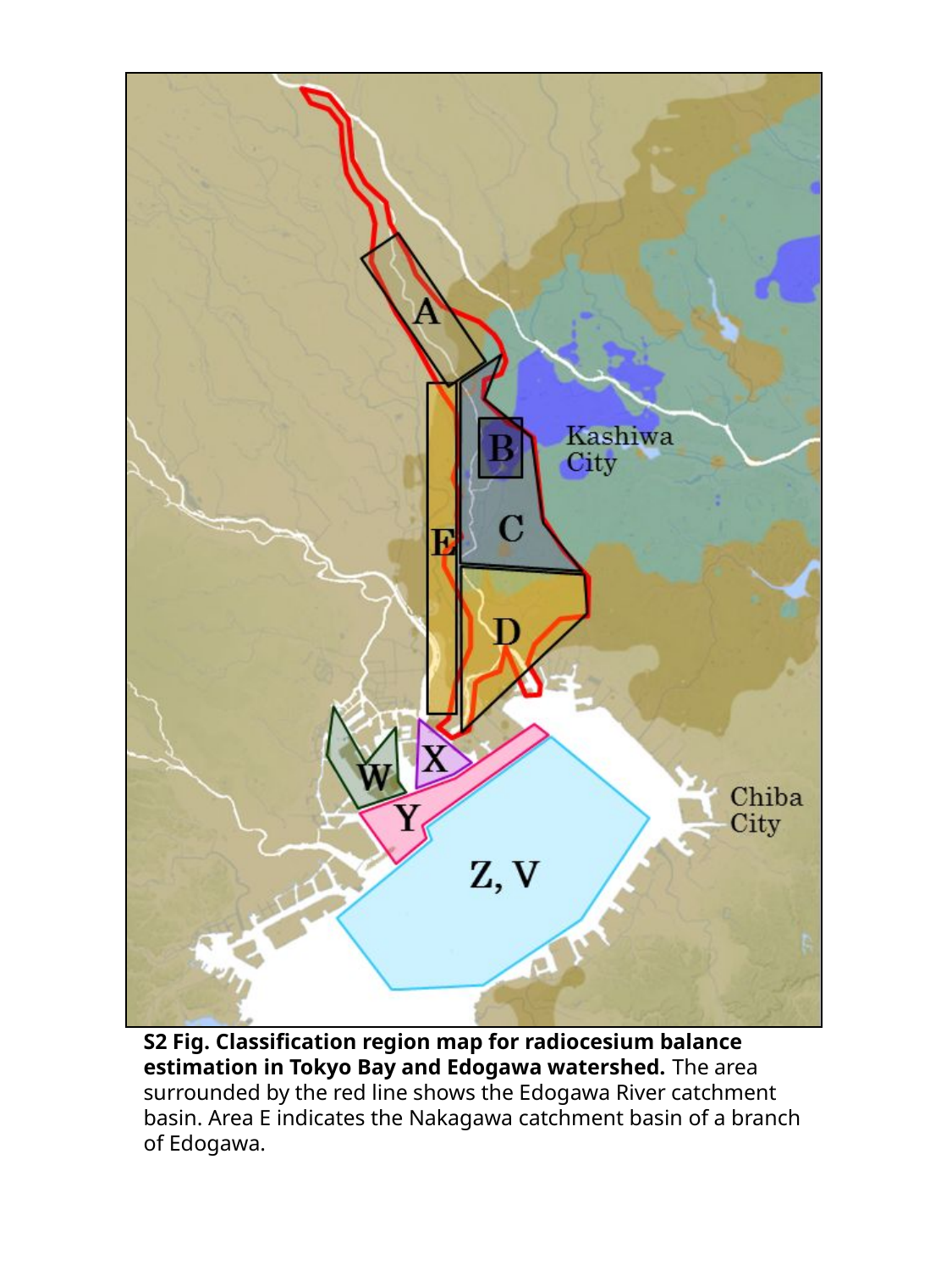

S2 Fig. Classification region map for radiocesium balance estimation in Tokyo Bay and Edogawa watershed. The area surrounded by the red line shows the Edogawa River catchment basin. Area E indicates the Nakagawa catchment basin of a branch of Edogawa.
