# Supplementary material for: Spatiotemporal distribution and fluctuation of radiocesium in Tokyo Bay in the five years following the Fukushima Daiichi Nuclear Power Plant (FDNPP) accident
Source: PLoS One. 2018 Mar 1;13(3):e0193414. doi: 10.1371/journal.pone.0193414 (PMC5832246; doi:10.1371/journal.pone.0193414)
Supplement: S3 Fig — The green arrow indicates the average particle diameter. (PPTX) [file pone.0193414.s003.pptx]

## Slide 1
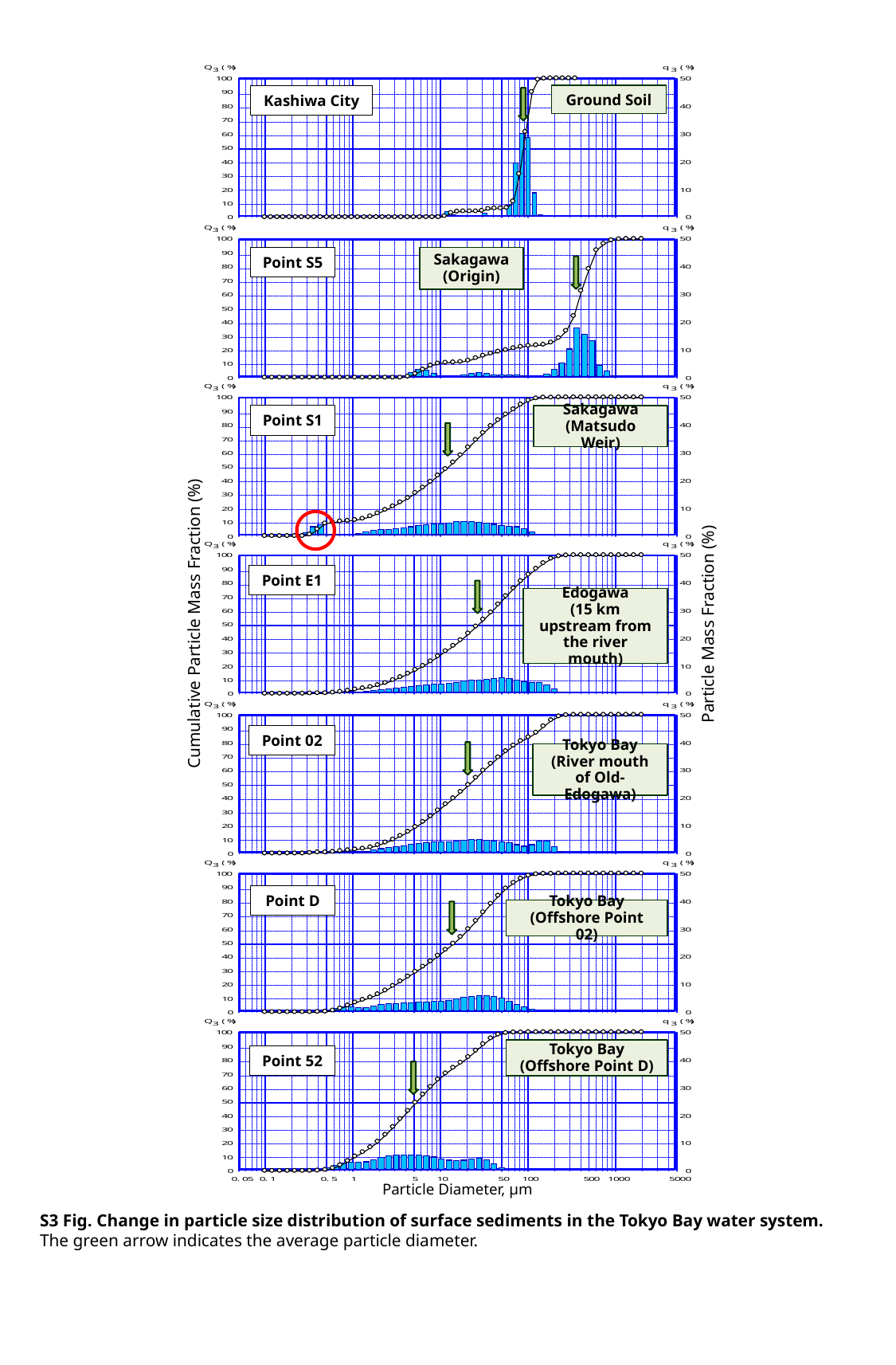

Kashiwa City
Ground Soil
Sakagawa
(Origin)
Point S5
Point S1
Sakagawa
(Matsudo Weir)
Point E1
Edogawa
(15 km upstream from the river mouth)
Cumulative Particle Mass Fraction (%)
Particle Mass Fraction (%)
Point 02
Tokyo Bay
(River mouth of Old-Edogawa)
Point D
Tokyo Bay
(Offshore Point 02)
Tokyo Bay
(Offshore Point D)
Point 52
Particle Diameter, μm
S3 Fig. Change in particle size distribution of surface sediments in the Tokyo Bay water system. The green arrow indicates the average particle diameter.
