# Supplementary material for: Spatiotemporal distribution and fluctuation of radiocesium in Tokyo Bay in the five years following the Fukushima Daiichi Nuclear Power Plant (FDNPP) accident
Source: PLoS One. 2018 Mar 1;13(3):e0193414. doi: 10.1371/journal.pone.0193414 (PMC5832246; doi:10.1371/journal.pone.0193414)
Supplement: S4 Fig — S: Sakagawa river, E: Edogawa and Old-Edogawa rivers, X: Old-Edogawa estuary, Y: Off the Old-Edogawa estuary, Z: Center of Tokyo Bay, V: Tamagawa estuary, W: Sumidagawa estuary. (PPTX) [file pone.0193414.s004.pptx]

## Slide 1
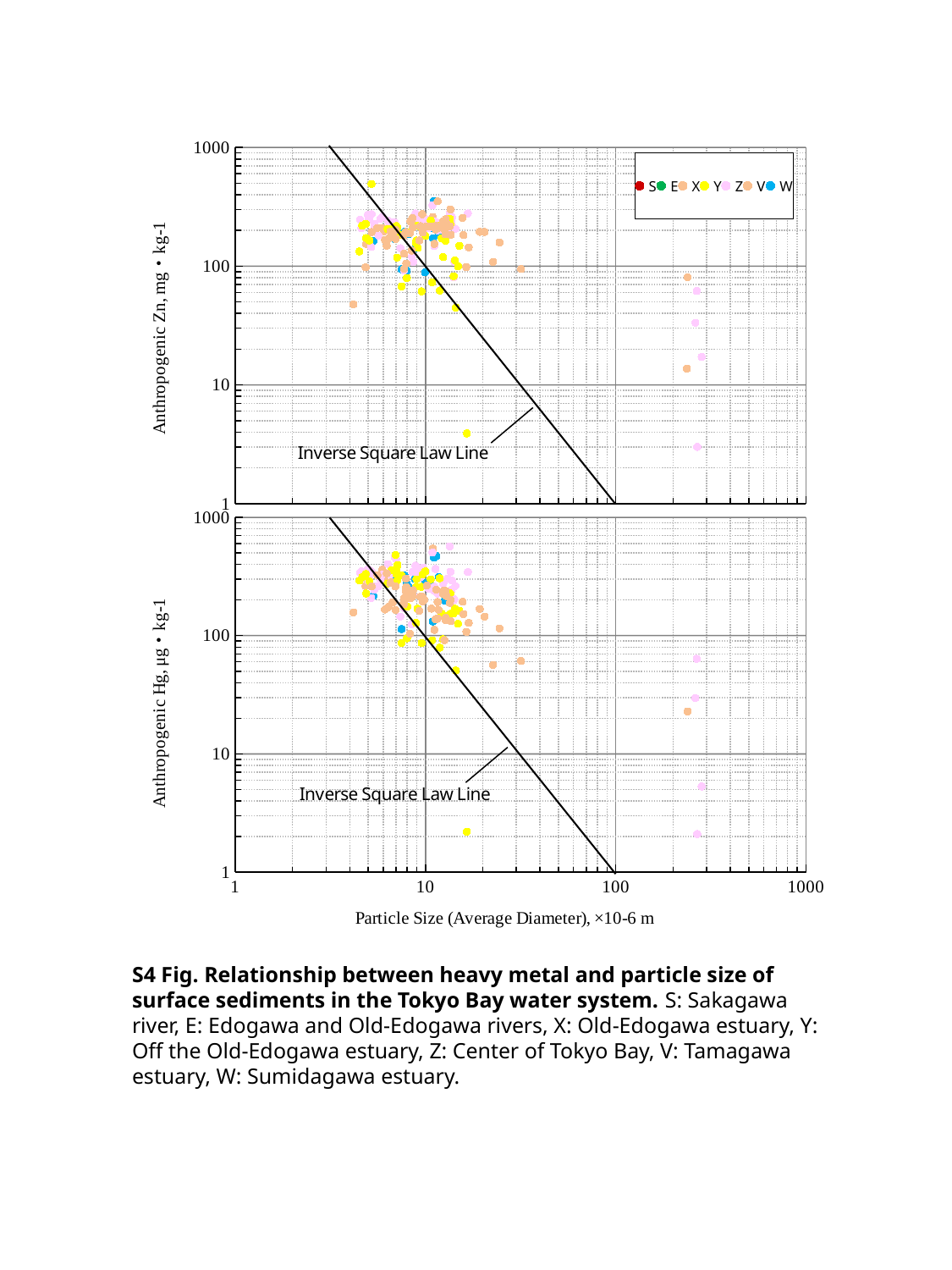

### Chart
| Category | | | | | | | |
|---|---|---|---|---|---|---|---|
### Chart
| Category | | | | | | | |
|---|---|---|---|---|---|---|---|S4 Fig. Relationship between heavy metal and particle size of surface sediments in the Tokyo Bay water system. S: Sakagawa river, E: Edogawa and Old-Edogawa rivers, X: Old-Edogawa estuary, Y: Off the Old-Edogawa estuary, Z: Center of Tokyo Bay, V: Tamagawa estuary, W: Sumidagawa estuary.
