# Supplementary material for: Spatiotemporal distribution and fluctuation of radiocesium in Tokyo Bay in the five years following the Fukushima Daiichi Nuclear Power Plant (FDNPP) accident
Source: PLoS One. 2018 Mar 1;13(3):e0193414. doi: 10.1371/journal.pone.0193414 (PMC5832246; doi:10.1371/journal.pone.0193414)
Supplement: S5 Fig — (a) Viewing the Tokyo metropolitan from Area X. (b) Core sampling by a diver. (c)-(e) Sediment core in seabed and the collected core sample. (f), (g) Surface sediment collected by an Ekman-Birge sampler. (PPTX) [file pone.0193414.s005.pptx]

## Slide 1
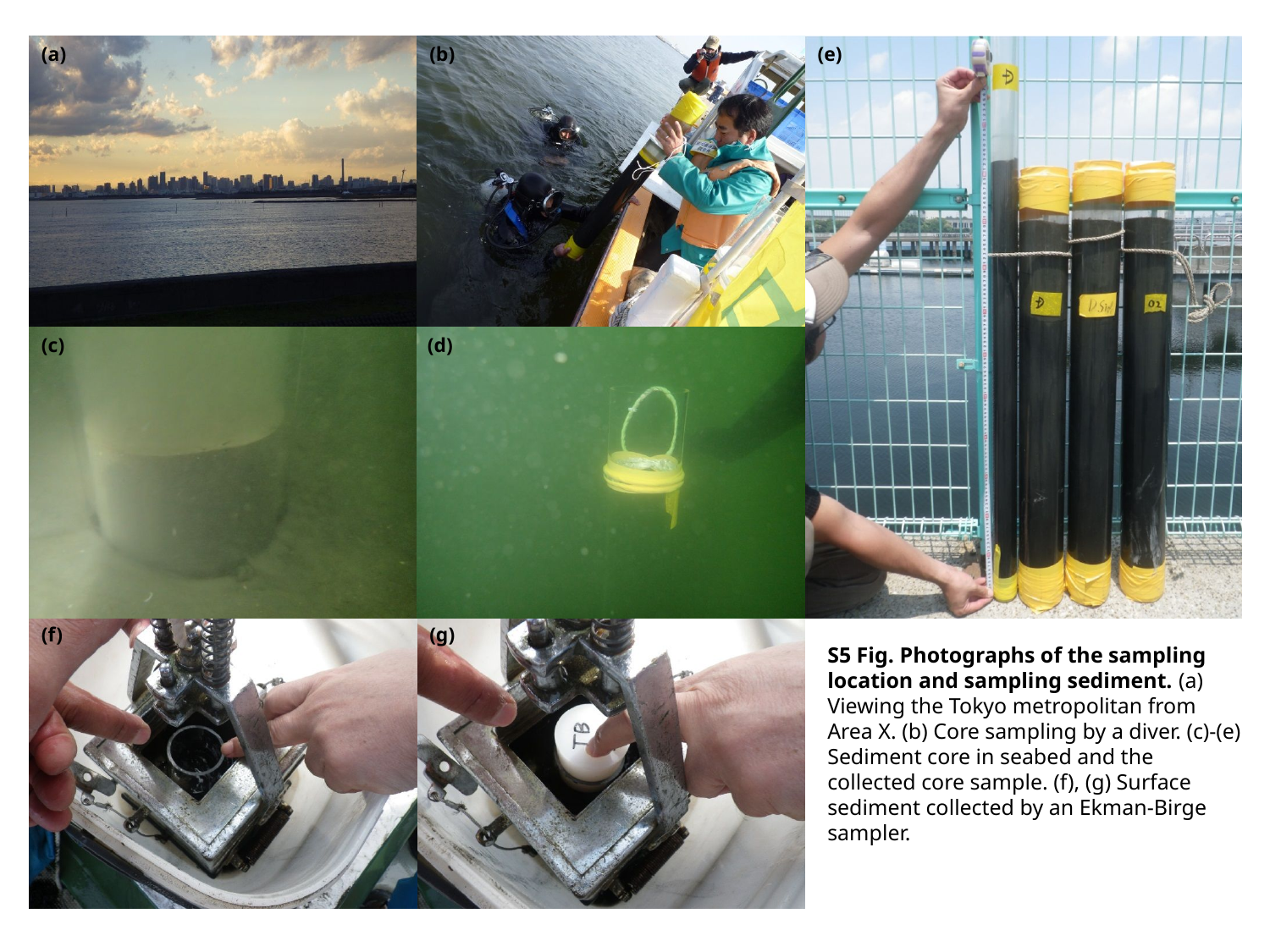

(b)
(a)
(e)
(d)
(c)
(g)
(f)
S5 Fig. Photographs of the sampling location and sampling sediment. (a) Viewing the Tokyo metropolitan from Area X. (b) Core sampling by a diver. (c)-(e) Sediment core in seabed and the collected core sample. (f), (g) Surface sediment collected by an Ekman-Birge sampler.
